# The Best for the Most Important: Maintaining a Pristine Proteome in Stem and Progenitor Cells

**DOI:** 10.1155/2019/1608787

**Published:** 2019-05-02

**Authors:** Bertal H. Aktas, Berin Upcin, Erik Henke, Manju Padmasekar, Xuebin Qin, Süleyman Ergün

**Affiliations:** ^1^Division of Hematology, Department of Medicine, Brigham and Women's Hospital and Harvard Medical School, 75 Francis Street, Boston, MA 02115, USA; ^2^Institute of Anatomy and Cell Biology, Julius-Maximilians Universität Würzburg, Koellikerstrasse 6, Würzburg, 97070 Bayern, Germany; ^3^Department of Neurology, Temple University, Philadelphia, USA

## Abstract

Pluripotent stem cells give rise to reproductively enabled offsprings by generating progressively lineage-restricted multipotent stem cells that would differentiate into lineage-committed stem and progenitor cells. These lineage-committed stem and progenitor cells give rise to all adult tissues and organs. Adult stem and progenitor cells are generated as part of the developmental program and play critical roles in tissue and organ maintenance and/or regeneration. The ability of pluripotent stem cells to self-renew, maintain pluripotency, and differentiate into a multicellular organism is highly dependent on sensing and integrating extracellular and extraorganismal cues. Proteins perform and integrate almost all cellular functions including signal transduction, regulation of gene expression, metabolism, and cell division and death. Therefore, maintenance of an appropriate mix of correctly folded proteins, a pristine proteome, is essential for proper stem cell function. The stem cells' proteome must be pristine because unfolded, misfolded, or otherwise damaged proteins would interfere with unlimited self-renewal, maintenance of pluripotency, differentiation into downstream lineages, and consequently with the development of properly functioning tissue and organs. Understanding how various stem cells generate and maintain a pristine proteome is therefore essential for exploiting their potential in regenerative medicine and possibly for the discovery of novel approaches for maintaining, propagating, and differentiating pluripotent, multipotent, and adult stem cells as well as induced pluripotent stem cells. In this review, we will summarize cellular networks used by various stem cells for generation and maintenance of a pristine proteome. We will also explore the coordination of these networks with one another and their integration with the gene regulatory and signaling networks.

## 1. Introduction

During early embryogenesis, inner cell mass of the embryo gives rise to pluripotent stem cells. They expand and commit to progressively restricted multipotent stem and progenitor lineages as embryonic development proceeds. The adult stem and progenitor cells, which descend from pluripotent stem cells, maintain tissue homoeostasis under physiological and pathophysiological conditions. Stem and progenitor cells as well as induced pluripotent stem cells (iPSCs) are considered to have an immense potential for cellular therapy of various human disorders.

Proteins are the master regulators and work horses of almost all cellular functions including DNA repair and replication, RNA and protein synthesis and quality control, energy generation, immune defense, maintenance of cellular homoeostasis, and cell division and death. Given the critical role of proteins for cellular functions, it is not surprising that organismal longevity is associated with and is dependent on the maintenance of a stable proteome [1]. Similarly, success of pluripotent stem cells in giving rise to a fully functional and reproductively enabled offspring as well as ability of the multipotent stem and progenitor cells to maintain tissue homoeostasis requires production and maintenance of an appropriate mix of error-free proteins. This is accomplished by coordinated activities of networks responsible for protein synthesis, folding, quality control, and degradation. These networks are integrated with gene regulatory and signaling networks, energy metabolism, and extracellular signaling cascades to minimize damage to existing proteome and maintain proper composition of proteins as demanded by the function of each cell.

The task of generating and maintaining a pristine proteome is particularly challenging because stem and progenitor cells must synthesize an appropriate mix of proteins necessary for all cellular functions, fold them correctly, protect them from damage, and remove unfolded, misfolded, damaged, or stage-specific proteins. Failure to maintain a pristine proteome is associated with a multitude of human disorders. In addition, restoration of tissue homeostasis after pathologic insults is fully dependent on the ability of adult stem and progenitor cells to self-renew and differentiate, which requires a pristine proteome. Similarly, patient-derived stem and progenitor cells and iPSCs are subject to various environmental insults during *in vitro* culture or at the site of implantation *in vivo*. The ability to maintain a pristine proteome is essential for expansion and differentiation of these cells upon transplantation. Therefore, there is a clear need to better understand the mechanisms underlying the generation and maintenance of a flawless proteome in the development, survival, and differentiation of pluripotent (embryonic) stem cells as well as adult stem and progenitor cells. Furthermore, understanding how the cellular networks responsible for maintaining a pristine proteome are integrated and interact with extrinsic and intrinsic cues to affect the maintenance of stemness, and differentiation will allow us to capture the full potential of various stem cell populations in regenerative medicine.

The goal of this review is to summarize our current understanding of how stem cell including ESCs, iPSCs, and various adult stem and progenitor cell types generate and maintain a pristine proteome. We will discuss how proteome maintenance is coordinated with self-renewal, commitment to downstream lineages, and terminal differentiation. We will pay particular attention to the roles of the cytoplasmic chaperome, integrated endoplasmic reticulum (ER) stress response (IERSR), ubiquitin proteasome system (UPS), and autophagy and their integration with one another and other signaling cascades in the generation and maintenance of a pristine proteome. Finally, we will review published data on how adult stem and progenitor cells maintain their proteome and on the pathophysiologic consequences of failure to maintain a pristine proteome. Due to space limitations, we cannot cover the rather extensive research in this area. However, we hope to convey sufficient information to those new to the field, particularly those faced with the challenges of maintaining, propagating, differentiating, and transplanting embryonic stem cells, adult stem and progenitor cells, and iPSCs.

## 2. Proteostasis in the Maintenance, Expansion, and Differentiation of Stem Cells

**Figure 1:**
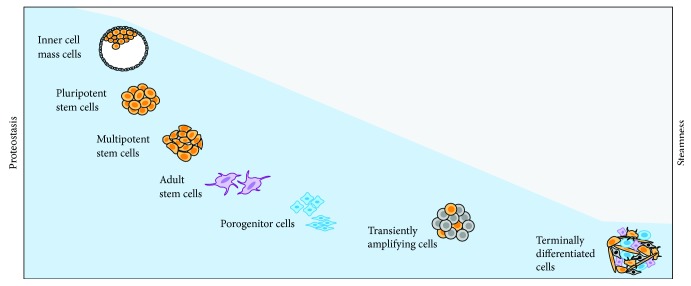
Pluripotent stem cells possess the most pristine proteome.

Pluripotent stem cells give rise to all the cells in the body. As such, any defects in the macromolecules of these cells can have profound consequences for the downstream lineages. As the first cells in the lineage, the pluripotent stem cells are different from lineage committed and other differentiated cells not only in their gene regulatory networks, epigenetic programming, and signaling pathways but also in the regulation of protein homoeostasis (proteostasis, reviewed by [2]). Pluripotent stem cells synthesize proteins at much higher rate than adult stem and progenitor or terminally differentiated cells. The transcription factor Nanog appears to be the master regulator of the small ribosomal subunit processome, which is responsible for 18S rRNA biogenesis [3, 4]. This is coupled with highly efficient translation of mRNAs coding for ribosomal proteins and ribosome biogenesis which appear to be essential for unlimited self-renewal and pluripotency, two critical traits of pluripotent stem cells. Consistently, inhibitors of protein synthesis or mutations that reduce the rate of protein synthesis cause a loss of pluripotency [3]. The high rate of the protein synthesis in pluripotent stem cells requires a highly active folding and processing machinery. This is particularly important because incorrectly folded or aggregated proteins will interfere with unlimited self-renewal [5]. Pluripotent stem cells can self-renew, at least in part, through asymmetric cell division [6]. In asymmetric division, mother cells are destined for immortality, keeping their pluripotency while daughter cells are destined to give rise to multipotent stem and progenitor lineages. To maintain immortality and pluripotency, the most pristine proteome is segregated to mother cells through yet to be fully understood mechanism that involves vimentin [7, 8]. Because almost all of the defective macromolecules are segregated to daughter cells, defective proteins, particularly those with half-lives on the order of days or even months/years, will concentrate in the multipotent stem and progenitor lineages [6, 9]. This has the potential to compromise tissue and organ development, which depends on the expansion and differentiation of lineage-restricted multipotent stem and progenitor cells. Thus, maintenance of a pristine proteome becomes critical not only for pluripotent stem cells' immortality but also for maintenance and proper functioning of adult stem and progenitor cells tasked with forming and maintaining tissues and organs [10–12]. Because of these reasons, pluripotent stem cells have very low tolerance to the accumulation of damaged, misfolded, and/or aggregated proteins (Figure 1). Consistently, these cells maintain a robust folding machinery and chaperone network as well as mechanisms for the surveillance and removal of unfolded/misfolded proteins [13]. Future studies should advance our understanding of the molecular networks responsible for generation and maintenance of pristine proteome and determine if they could be modified for realizing the potential of stem cells for alleviating human suffering.

### 2.1. The Molecular Machinery of Proteostasis

Generation and maintenance of a pristine proteome is dependent on the integration of cellular machineries that (i) synthesize new proteins, (ii) properly fold newly synthesized proteins, (iii) survey the proteome for and mark improperly folded, aggregated, or damaged proteins, (iv) transport and degrade improperly folded proteins, and (v) coordinate these processes with each other and with other intra- and extracellular networks to maintain a stable proteome. One example of coordinating protein synthesis, folding, and degradation with other cellular processes by pluripotent stem cells is their preference for glycolysis over oxidative phosphorylation even though the former is energetically less efficient (reviewed by [10]). The metabolic preference of pluripotent stem cells for glycolysis reduces free radical generation by oxidative phosphorylation thereby reducing damage to their proteins and other macromolecules.

**Figure 2:**
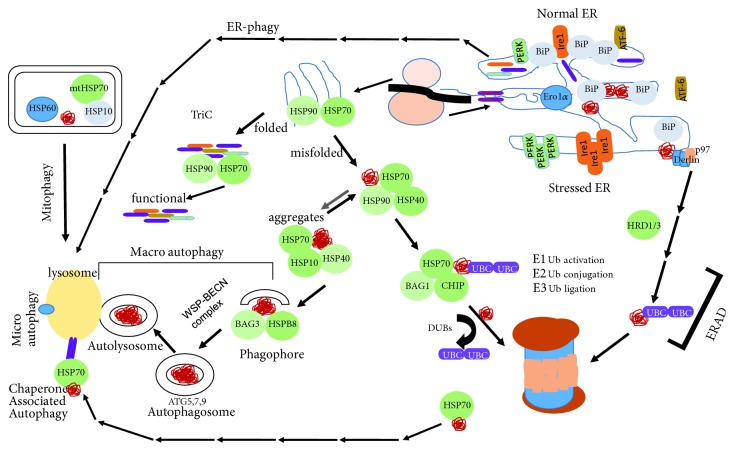
Schematic depiction of protein synthesis, folding and degradation in stem cells.

Proteins are synthesized by free cytoplasmic ribosomes or endoplasmic reticulum- (ER-) associated ribosomes. Most cytoplasmic proteins are synthesized by free cytoplasmic ribosomes and folded and processed by cytoplasmic chaperones (Figure 2) [14, 15]. Proteins destined for secretion, localization to plasma membrane, or other organelles are usually synthesized by ER-associated ribosomes and cotranslationally transported into ER lumen where they are folded, glycosylated, and further modified before transfer to trans-Golgi (Figure 2) [16–19]. They are further modified and packaged in Golgi cisternae for transport to their destination. Unfolded, misfolded, damaged, and/or aggregated proteins are eliminated through the ubiquitin/proteasome system (UPS) or by autophagy-mediated degradation depending on the size of the protein complex and whether they are integral to an organelle (Figure 2) [20–23].

#### 2.1.1. Protein Folding in the Cytoplasm: Chaperones and Folding Machinery

Protein folding requires aid of specialized proteins, cofactors, and chaperones. The human chaperome consists of ~350 proteins that differ in their functionality and intracellular localization [24]. The chaperones are grouped into the functionally nonoverlapping families such as the heat-shock proteins (HSPs) of 90 kDa (HSP90s), 70 kDa (HSP70s), 60 kDa (HSP60s), 40 kDa (HSP40s), and small HSPs [24–26]. Chaperones are involved in proteome biogenesis and play essential roles in protecting cells from proteotoxic stress [16]. Protein folding is tightly coupled to mRNA translation through a process termed cotranslational folding [15]. Several cofactors such as ribosome-associated complex (RAC) interacts with the ribosome-binding HSP40 cochaperone complex and nascent polypeptide-associated complex (NAC), a dimeric complex of a and b subunits that associates with ribosomes and plays critical roles in cotranslational folding (reviewed by [18]). The HSP70 system also plays a critical role in this process; multiple HSP70 family proteins associate with a nascent polypeptide chain to prevent unproductive interdomain interactions and facilitate cotranslational folding [27]. HSP70 also interacts with HSP90 and chaperonins such as T-complex protein-1 ring complex (TRiC/CCT) to transfer substrates in need of more specialized folding (Figure 2) [28, 29]. TRiC/CCT, which belongs to the HSP60 family of chaperones, folds about 10% of cellular proteins including cytoskeletal proteins and G-protein-coupled receptors [30]. In addition to its role in protein folding, HSP90 also plays an important role in the maturation and maintenance of regulatory proteins such as kinases, steroid hormone receptors, and transcription factors [31]. Important in the context of pluripotent stem cell maintenance is the demonstration that HSP90 binds to pluripotency factors Oct4 and Nanog, protecting them from UPS-dependent degradation [13].

Heat-shock proteins and components of the TRiC/CCT complex and other chaperonins are expressed at much higher level in the pluripotent stem cells compared to multipotent and lineage-committed progenitor cells [20, 32–36]. Molecular and chemical genetic studies demonstrate that an intact chaperone network is essential for self-renewal and pluripotency. The TRiC/CCT complex appears to be essential for pluripotency and self-renewal as well as for the survival of pluripotent stem cells. Partial knockdown of various CCT subunits suppresses the expression of pluripotency markers and induces the expression of germ layer markers in human ESCs and iPSCs, while their complete knockdown causes death of hESC and iPSC cells [36]. Similarly, reducing the expression and/or the activity of HSP90 or its cochaperone HOP causes loss of pluripotency and differentiation of ESCs [13]. Like most other HSPs, the HSP70 family of chaperones is also expressed at much higher level in pluripotent stem cells compared to lineage-committed, differentiating, or terminally differentiated cells. These include HSP8A, HSPA1A, HSPA1B, HSP5A (BiP, localized in the ER), and HSP9 (mortalin, localized to mitochondria). These proteins appear to inhibit apoptosis and promote self-renewal [35, 37]. It would be interesting to determine whether HSPs' relative abundance, alone or in combination with some other molecules, can be used as a marker to distinguish pluripotent or multipotent stem cells from lineage-restricted stem cells or lineage-committed progenitor cells.

#### 2.1.2. Protein Folding and Quality Control in the Endoplasmic Reticulum (ER)

The endoplasmic reticulum (ER) is the organelle tasked with folding, glycosylating, and exporting secreted and membrane-targeted proteins [38, 39]. The ER is also an organelle that responds to and propagates various intracellular and extracellular signals [40]. Peptide growth factors and hormones generate second messengers such as phosphatidylinositol bisphosphate (PIP2) through their cognate cell surface receptors. PIP2 activates signal transducers located on the ER membrane to release Ca^++^ from ER stores, which results in the activation of Ca^++^-regulated signaling nodes [41, 42]. The ER is also a synthetic organelle where much of the fatty acid and lipid biosynthesis takes place [43].

As a synthetic, processing, and signaling organelle, ER homeostasis is influenced by a multitude of extracellular and intracellular factors. The proper functioning of the ER is required for maintaining a pristine proteome. Just like the proteins synthesized by cytoplasmic free ribosomes, translation and folding of proteins synthesized by ER-associated ribosomes are coupled in a process termed cotranslational processing [44]. Nascent polypeptides are recognized by signal recognition particle and threaded through the Sec61 channel into the ER as they emerge from ribosome. In the reductive environment of ER, they are folded with the help of ER chaperones, foldases, and isomerases [45]. The first step in the ER quality control is the detection of misfolded, unfolded, or aggregated polypeptides, collectively termed defective ribosomal products [46]. Such polypeptides are redirected to the cytoplasm for degradation, usually by the UPS [47].

Maintenance of pristine proteome also depends on matching the capacity of the ER with the demand for protein folding. When this balance is perturbed, unfolded and/or misfolded proteins accumulate within the lumen of the ER, causing ER stress (Figure 2). ER stress is usually resolved through activation of the three interrelated canonical trans-ER-membrane signaling nodes controlled by PKR-like endoplasmic reticulum kinase (PERK), inositol-requiring enzyme 1 (Ire1), and activating transcription factor 6 (ATF-6) [48, 49]. The canonical ER stress-response signaling, also termed integrated endoplasmic reticulum stress response (IERSR), inhibits translation to reduce the demand for protein folding, induces the expression of ER chaperones and ER biogenesis genes to expand the ER's folding capacity, and induces the expression of the proteins that undertake the retrograde transport and degradation of unfolded proteins from the ER (ERAD) to resolve ER stress [50–52]. The IERSR signaling engenders a transcriptional, translational, and posttranslational gene expression program that regulates cell proliferation, differentiation, senescence, or death in a context and cell type-dependent manner (Figure 2).

PERK phosphorylates the alpha subunit of the eukaryotic translation initiation factor 2 (eIF2*α*) on serine 51. This phosphorylation has the dual effect of suppressing general translation while paradoxically inducing translation of a subset of mRNAs. These include mRNAs coding for transcription factor ATF-4 and C/EBP homologous DNA damage inducible protein (CHOP) [53, 54]. ATF-4 and CHOP cooperate with other transcription factors to increase the folding capacity of the ER, activate ERAD, and induce autophagy to restore ER homoeostasis [55]. If all of these fail to restore ER homoeostasis, CHOP will induce cell death. PERK also directly phosphorylates and activates nuclear factor erythroid-like 2 (Nrf2) gene product and by yet to be fully understood mechanism activates NF-*κ*B [56–59]. These two transcription factors play critical roles in cell survival under stress. PERK also impinges on microRNAs, lipid metabolism, and vascular endothelial growth factor (VEGF) signaling [60–65]. PERK-deficient embryos develop normally except for the progressive demise of the endocrine pancreas, which results in early onset diabetes and premature death [66]. This phenotype resembles human Wolcott-Rallison syndrome where compound PERK mutations cause very early onset diabetes and early childhood death [67, 68]. These data indicate that while it is important for normal development and healthy life, PERK signaling is dispensable for generation, maintenance, self-renewal, and differentiation of pluripotent stem cells. Relevance of PERK signaling for maintenance and normal function of adult stem and progenitor cells will be discussed later.

Ire1, a dual endonuclease and kinase, plays essential roles in adaptation to ER stress. Ire1-catalyzed cytoplasmic splicing of X-box binding protein 1 (Xbp-1) mRNA causes a frame shift at the splice junction. This generates an Xbp-1 mRNA isoform that codes for a transcriptionally active protein, Xbp-1 spliced (Xbp-1S) [69]. Xbp-1S induces transcription of mRNAs coding for chaperones, ER biogenesis, and ERAD proteins as well as its own and CHOP mRNA [70]. Under overwhelming stress, Ire1 also splices other mRNAs leading to their degradation, in what is termed regulated Ire1-dependent decay (RIDD) that can further reduce demand on the ER for protein folding [71–73]. Ire1, through its kinase domain, forms a complex with and activates TNF-associated factor 2 (TRAF-2), which in turn activates the apoptosis signal-regulated kinase 1 (ASK1). ASK1 induces apoptosis through c-jun N-terminal kinase (JNK) [74]. Deletion of Ire1 causes placental defects and consequent embryonic failure while deletion of Xbp-1 causes embryonic lethality due to defective erythropoiesis in the liver secondary to liver hypoplasia [75]. The fact that the Ire1- and Xbp-1-deficient embryos develop near normal until midpregnancy indicates that Ire1 and Xbp-1 are dispensable for self-renewal, pluripotency, and differentiation of pluripotent stem cells into multipotent stem and progenitor cells and differentiation into most tissues [76, 77]. Currently, there are no animal models of dual PERK and Ire1 deficiency; therefore, it is not possible to determine if simultaneous ablation of both signaling nodes will interfere with the establishment and/or maintenance of pluripotent stem cells.

ATF-6 is the third member of the canonical ER stress-response network. Under ER stress, ATF-6 translocate to Golgi where it is cleaved by site-1 and site-2 specific proteases releasing transcriptionally active cytoplasmic domain. The cleaved ATF6 binds to promoters containing ER stress response elements (ERSE) and to conserved GRP promoters. Transcription from these promoters induces the expression of ER chaperones including GRP78, GRP94, calnexin, XBP-1, and calreticulin [5, 78, 79]. Either a or b homologs of ATF-6 can be deleted individually with no consequence for animal development; however, simultaneous deletion of both homologs is embryonic lethal at the somite stage [80, 81]. This indicates that ATF-6 is not essential for pluripotency, self-renewal, and differentiation of pluripotent stem cells into multipotent stem and progenitor cells and differentiation into three germ layers up to early somite stage but is essential for further development [80, 81].

While eliminating activity of any of the three ER stress-response pathways does not interfere with the generation and survival of pluripotent stem cells, lineage commitment and early differentiation, deletion of an ER stress sensor protein, binding immunoglobulin protein (BiP) also known as GRP78, does indeed interferes with the expansion of inner cell mass into pluripotent stem cells and causes preimplantation embryonic lethality [82]. BiP binds to and locks Ire1, PERK, and ATF-6 into inactive heterodimers. It is thought that unfolded proteins in the ER display exposed hydrophobic domains that bind BiP [40]. When the levels of these exposed hydrophobic domains increase, they titrate BiP away from PERK, Ire1, and ATF-6. Free PERK and Ire1 form homodimer and/or multimers, while free ATF-6 moves to trans-Golgi where its cytoplasmic domain is cleaved by site 1 and 2 specific proteases, which then translocate to the nucleus (Figure 2) [48, 49].

While studies with deletion of BiP suggest that sustained activation of all three branches of the IERSR without inhibitory feedback will cause demise of pluripotent stem cells, it is unclear whether this would indeed happen during normal development, because physiologic IERSR caused by unfolded proteins is reversed once ER homeostasis is reestablished. In contrast, complete deletion of BiP could unleash a massive IERSR with no apparent rectifying mechanisms, potentially locking the IERSR permanently to “on” position. This may generate a vicious cycle wherein the IERSR causes oxidative stress and secondary and tertiary transcriptional and translational changes that trigger mitochondrial and nucleolar stress and eventual cell death [83, 84]. Furthermore, BiP is also involved in translocation of proteins into ER, Ca^++^ homoeostasis, and can function as a signaling molecule when located on the cell membrane [85]. Nevertheless, these data offer significant clues as to the consequences of simultaneous sustained activation of all three nodes of the IERSR, which may occur under some pathologic conditions.

Interestingly, complete deletion of GRP94, the ER-resident HSP-90 family member, also causes embryonic lethality but much later during development [86]. These embryos have already formed germ layers indicating that GRP94 is not required for maintenance and differentiation of pluripotent stem cells but apparently required for proper embryogenesis beyond this stage. Consistently, GRP94 deficiency does not inhibit the proliferation of mouse ESCs (mESCs) in culture or their differentiation into germ layers; however, GRP94-deficient cells cannot differentiate into cardiac, smooth muscle, or skeletal muscle tissues [87]. Better understanding of the role of the IERSR in the development and differentiation of pluripotent and multipotent stem cells may pave the way for the utilization of molecularly targeted IERSR modifiers that would improve the potential of stem and progenitor cells in tissue repair and regeneration.

The IERSR is not restricted to restoring ER homoeostasis upon the accumulation of unfolded proteins but also interacts with other signaling cascades to regulate various cellular functions. Recent studies indicate that the IERSR is activated during lineage commitment and differentiation of pluripotent stem cells. For example, chemical induction of the IERSR in mESC-derived embryoid bodies or in mESC monolayer cultures induces an endoderm-specific gene expression program that leads to differentiation of mESC into definitive endoderm [88]. This is accomplished by activating TGF-*β*/smad2 signaling and their downstream effectors. Induction of the IERSR signaling in mESC also suppresses the activity of GSK3*α*/*β*, which results in the stabilization and nuclear accumulation of *β*-catenin [88]. The Wnt/*β*-catenin complex turns on a gene expression program that appears to be essential for the differentiation of mESC into definitive endoderm. Consistently, inhibition of Wnt/*β*-catenin signaling interferes with the differentiation of mESC into definitive endoderm. Taken together, these data indicate that the IERSR signaling activates the TGF-*β*/smad2 and Wnt/*β*-catenin pathways to induce the differentiation of ESCs into definitive endoderm [88].

### 2.2. Protein Degradation in Regulating Pluripotency and Lineage Commitment

Similar to protein synthesis and folding, degradation of unfolded, misfolded, or otherwise damaged proteins is also required for the maintenance of a pristine proteome. Furthermore, disposal of stage-specific proteins is essential for normal functioning of cells. For example, several cell cycle proteins must be degraded to allow for cell cycle transitions. Protein degradation therefore plays a critical role in maintaining a pristine proteome as well as an appropriate mix of proteins required for responding to intracellular signals and external stimuli [89]. Two proteolytic pathways that play essential roles in proteostasis are the ubiquitin proteasome system (UPS) and autophagy. We will now discuss these pathways in more detail.

#### 2.2.1. The Ubiquitin Proteasome System (UPS)

The UPS is the major pathway for the removal of proteins, whether they are damaged or no longer needed. It involves marking proteins by attaching a polyubiquitin chain to their lysine residues and transferring polyubiquitinated proteins to the proteasome for degradation. In the first step of ubiquitination, a small protein called ubiquitin is activated by ubiquitin-activating enzyme (E1) and subsequently transferred to ubiquitin-conjugating enzymes (E2s). A third set of enzymes, E3 ligases, transfer this ubiquitin to target proteins [90, 91]. These three steps are repeated to add additional ubiquitin molecules to the primary ubiquitin to form a polyubiquitin chain (Figure 2). There are more E2 ligases than E1 ligases and dramatically more E3 ligases than E1 and E2 ligases combined. This is because UPS owes its specificity and selectivity to substrate recognition by E3 ligases. Not surprisingly, the repertoire of E3 ligases and their regulators shows significant differences between pluripotent stem cells and differentiated cells [20, 92, 93]. Remarkably, several pluripotency factors including Oct4 and Nanog can be degraded by UPS. The polyubiquitinated proteins are recognized and degraded by the proteasome which is formed by the assembly of 20S catalytic core and the 19S regulatory subunit [94].

Consistent with the need to maintain a pristine proteome, both hESCs and mESC display higher proteasome assembly and activity than differentiated cells [20, 93]. Given their need for higher UPS activity, ESCs are extremely sensitive to proteasome inhibition, even a mild downregulation of the proteasome results in increased differentiation and decreased levels of pluripotency factors [20, 95]. The forkhead transcription factor FOXO4 promotes the expression of PSMD11/RPN6, a 19S scaffolding subunit that stimulates the interaction of 19S regulatory complex with the 20S catalytic core [20, 96]. Stimulation of UPS by FOXO4 appears to be necessary for the differentiation of ESCs into neuronal cells [97]. Other components of the UPS that appear to be differentially expressed in ESCs include the deubiquitinating enzyme Psmd14 and the proteasome maturation protein (POMP) [93, 98]. Nrf2, which is directly phosphorylated by PERK, is responsible for high level of POMP expression. As a proteasome chaperone, POMP is not only important for the self-renewal of ESCs but also cellular reprogramming [98]. Degradation of damaged proteins through UPS is also essential for the differentiation of ESCs. Huwe1 is an E3 ligase that polyubiquitinates N-Myc (an important transcription factor for ESC maintenance) thereby playing a critical role in neural differentiation of mESCs [99].

Immunoproteasome, a variant of the proteasome, reportedly plays a critical role in differentiation as well. In the initial stages of differentiation, ESCs trigger termination of damaged proteins by induction of the catalytic subunit *β*5i (PSMB8) and the immunoproteasome regulatory activator PA28*αβ* [100, 101]. UPS regulates the level of Nanog, Oct-4, and c-Myc, which are critical pluripotency factors for natural as well as induced PSCs. Whether components of UPS can be modified to improve reprogramming of iPSCs and encourage these and natural pluripotent and/or multipotent stem cells towards lineage-restricted differentiation program remains to be determined.

#### 2.2.2. Autophagy: Titanic-Scale Proteome Maintenance

Autophagy is degradation of damaged or outlived organelles, macromolecules, and other cytosolic fractions too large to be handled by UPS. In autophagy, the engulfment of protein aggregates, organelles, or cytosolic pieces by a double membrane vesicle generates an autophagosome (Figure 2), which then fuses with the lysosome leading to degradation of its contents. Autophagy has a dual purpose of maintaining cellular homoeostasis while generating building blocks for anabolic processes. It plays an essential role in development, differentiation, or cellular reprogramming [102–105]. Protein degradation in the lysosome generates free amino acids, small di- and tri-peptides, and larger peptides that are released into the cytosol to be further metabolized [104]. In addition to functioning as a recycling pathway, autophagy also preserves cellular homeostasis by controlling the quality of proteins and organelles and degrading misfolded and aggregated proteins [26, 106]. Similar to UPS, autophagy can also selectively degrade proteins thereby regulating their relative abundance (Figure 2) [106–109].

Autophagy is regulated by the autophagy-related gene (ATG) products. These include ULK1, ATG13, FIP200, and ATG101, collectively known as ULK complex that forms a double membrane structure, phagophore, which is expanded by VPS34–BECN1 complex composed of VPS34, BECN1, AMBRA1, and ATG14L [106]. In another form of autophagy called chaperone-mediated autophagy (CMA), cytoplasmic proteins are recognized by chaperones such as HSP70 through a consensus sequence and transferred to the lysosome for proteolysis (Figure 2) [110].

### 2.3. Coordination of Protein Synthesis, Folding, Quality Control, and Degradation

Stem cells must coordinate protein synthesis, folding, quality control, and degradation to minimize energy expenditure, maintain an appropriate mix of proteins, and a pristine proteome. Earlier, we discussed how synthesis, folding, and degradation of cytoplasmic proteins are integrated. We will now discuss in some detail how these processes are coordinated for proteins synthesized by ER-associated ribosomes and processed in the ER.

**Figure 3:**
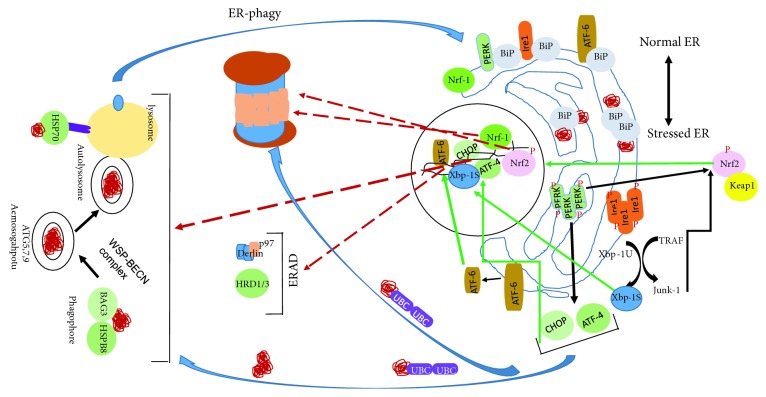
Integration of the integrated endoplasmic reticulum stress response (IERSR), ubiquitin proteasome system (UPS), and autophagy in maintaining a pristine proteome. The IERSR activates transcription factors that govern the expression of ER homoeostasis, UPS, and autophagy genes. Autophagy and UPS in turn degrade signaling molecules activated by the IERSR and trim ER and mitochondria to help these organelles return to their normal size and composition once unfolded proteins are cleared.

The presence of unfolded/misfolded proteins in the ER is the result of imbalance between demands for protein folding vs. the capacity of ER to fold client proteins, which trigger the IERSR. Upon activation of the IERSR, ATF-6 and to a lesser extent Xbp-1 and ATF-4 transcriptionally activate ERAD genes (Figure 3) [111–113]. The unfolded, misfolded, or aggregated proteins in the ER lumen are recognized by the coordinated action of ERAD proteins and retrotranslocated across the ER membrane by several protein complexes including Sec61 channel, Derlins, and Hrd1 ubiquitin ligase [114–116]. Almost all ERAD substrates are polyubiquitinated on the cytosolic side of the ER membrane by ubiquitin ligase complexes containing Hrd1, Hrd3, Usa1, Derlins, and other proteins. The IERSR also induces preemptive quality control machinery that through interactions of Derlins with signal recognition particle reroutes the ER-destined proteins to the cytoplasm where they are ubiquitinated with contribution from Bag6 and the AAA-ATPase p97 and degraded through UPS (Figure 3) [45].

Under physiologic conditions, capacity of the UPS exceeds demand for protein degradation. However, both ER and cytoplasmic stress significantly increase the demand on UPS. Expression of UPS proteins is controlled by Nrf1 and to a lesser extent Nrf2. PERK directly phosphorylates and thereby liberates Nrf2 from Keap1. Keap1 maintains Nrf2 in inactive state in the cytoplasm and instigates its degradation by UPS [57]. Another kinase that phosphorylates Nrf2 is c-jun N-terminal kinase (JNK) itself activated by Ire1. Phosphorylation of Nrf2 and its subsequent liberation from Keap1 enables nuclear accumulation of Nrf2 resulting in increased transcription of its target genes [117]. By inducing major antioxidant genes while reducing CHOP expression, Nrf2 promotes survival of stressed cells. Nrf2 also induces the expression of several chaperones, ERAD genes, ubiquitin ligases, and subunits of the proteasome complex, all of which play critical roles in the transport and degradation of unfolded, misfolded, or otherwise defective proteins (Figure 3) [118–120].

Nrf1 is an ER membrane anchored protein that translocates to the nucleus following deglycosylation and perhaps proteolytic cleavage and plays a major role in inducing the expression of almost all proteasome subunits [121–123]. Nrf1 activation is particularly strong in cells treated with proteasome inhibitors but is also pronounced in cells treated with tunicamycin, a glycosylation inhibitor that causes ER stress [124, 125]. While the molecular underpinnings of Nrf1 activation are not well understood, based on the robust activation of this transcription factor by proteasome inhibitors, it is likely that accumulation of polyubiquitinated client proteins in excess of UPS' capacity generates the signal(s) that activate Nrf1 (Figure 3).

Macro, micro, and chaperone-mediated autophagy interact with cellular pathways including cytoplasmic chaperones, IERSR, and oxidative stress (Figure 3) [126–128]. The PERK/eIF2*α*-phosphorylation arm of the IERSR induces core autophagy genes and cytosolic cargo receptors such as Map1lc3b (LC3B), Atg5, Atg3, Atg7, Atg10, Atg12, Atg16l1, Becn1, Gabarap, Gabarapl2, p62, and Nbr1 [129]. ATF-4, a downstream effector of phosphorylated eIF2*α*, transcriptionally upregulates REDD1, which results in the activation of tuberous sclerosis complex (TSC) that inhibits mTORC1. Suppression of mTORC1 activity leads to the activation of autophagy. Another effector of the IERSR, CHOP activates tribbles-related protein 3 (TRB3) that directly inhibits Akt, resulting in TSC activation, inhibition of mTORC1 activity, and thereby induction of the ULK1/2 complex. CHOP also upregulates ERO1-*α* transcription, which releases Ca^++^ from internal stores, activates calcium-calmodulin-dependent kinase II (CAMKII) that in turn activates ULK1/2 complex through inhibition of mTORC1. Last but not the least, ATF-4 and CHOP directly induce the transcription of key autophagy genes LC3, ATG12, and ATG5 [130–135]. Ire1 also induces autophagy by activating JNK through TRAF2 [136]. Autophagy in turn helps in reestablishing ER homoeostasis by trimming ER as part of recovery from stress [84]. Mammalian cells contain several autophagy receptors such as FAM134B, SEC62, RTN3, and CCPG1. These receptors play a critical role in sequestering isolated ER fragments into the lumen of autophagosome in which ER fragments with a delimiting membrane are digested (Figure 3) [137, 138].

### 2.4. Proteostasis in the Self-Renewal, Survival, and Differentiation of Adult Stem/Progenitor Cells

Adult stem cells and progenitor cells play critical roles in tissue homeostasis and repair, particularly following pathologic insults. These cells differ from the pluripotent stem cells in that they can give rise to many but not all tissues or cell types. Like pluripotent stem cells, adult stem/progenitor cells must self-renew and differentiate. These cells have developed various strategies to protect their macromolecules and organelles from damage because adult stem/progenitor cells must be preserved for the life of the organism. Adult stem and progenitor cells in different tissues are subject to different environmental cues and various levels of demand for tissue regeneration. Certain tissues such as small intestine epithelium and hematopoietic cells must be constantly replenished, more so under pathological insults. On the other hand, cardiac or neuronal stem and progenitor cells may be called upon to regenerate tissue only under pathologic insults; therefore, these cells may face lower demand in adult organisms. Not surprisingly, the strategies and dominant pathways used for proteome maintenance differ among different stem/progenitor cell types. Below, we will discuss strategies used by different stem and progenitor cells for maintaining a pristine proteome and how disruption those pathways affect self-renewal and differentiation of adult stem cells.

#### 2.4.1. Proteostasis in Neuronal Stem Cells' (NSCs) and Neuronal Progenitor Cells' Survival, Self-Renewal, and Differentiation

NSC self-renew and differentiate into neurons, astrocytes, and oligodendrocytes [139, 140]. Both autophagy and IERSR signaling play important roles in the self-renewal of NSCs [141–143]. In mammals, cortical neurogenesis can proceed through either direct neurogenesis, asymmetric division of the apical progenitors followed by differentiation, or indirect neurogenesis, generation of intermediate progenitors that can proliferate and differentiate into projection neurons. Activation of the PERK-eIF2*α*-ATF4 signaling interferes with generation of intermediate progenitors thus promoting direct neurogenesis while knocking down ATF-4 favors indirect neurogenesis [144]. These findings suggest that physiological IERSR signaling may play a role in cortical development. Consistently, mice expressing mutant BiP protein display a disordered pattern of layer formation as well as migration defects in the cerebellum [145]. Mutant BiP-expressing mice also display microcephaly in the cerebral cortex indicating that BiP regulates other crucial factors in brain development and highlights the role of proteostasis in mammalian neuronal development [146–148].

In adults, the subgranular zone of the hippocampal dentate gyrus is the major site of active neurogenesis [141, 142, 149]. The IERSR has been implicated in the viability of NSCs located in the subventricular zone (SVZ) of the lateral ventricle and the subgranular zone of the adult hippocampal dentate gyrus [141, 142]. Similarly, deletion of a single copy of critical autophagy genes Ambra1 or Beclin1 significantly reduces the self-renewal potential of NSCs in the SVZ while increasing the rate of apoptosis [143]. These data indicate that basal autophagy flux is essential for self-renewal and survival of neuronal stem and progenitor cells and likely in the early phases of their differentiation.

Neuronal differentiation is associated with increased PERK signaling [139]. Two chemical inducers of ER stress, tunicamycin and thapsigargin, enhance the differentiation of mESCs into neurons while inhibiting their differentiation into glial cells [150]. These agents transiently activate all three nodes of the canonical IERSR signaling; it is therefore not possible to determine which of these pathways is/are responsible for induction of neurogenesis. Suppressing the expression of HRD1, an E3 ubiquitin ligase that plays an important role in ERAD abrogates tunicamycin-induced neuronal differentiation [150]. Genetic suppression of ERAD by expression of mutant HRD1 may lead to sustained activation of all three IERSR signaling nodes, which would negate the beneficial effects of tunicamycin-induced transient IERSR [151–153]. Alternatively, recovery from stress or activation of ERAD/UPS may generate differentiation signals for neurogenesis.

Consistent with the role of the IERSR in neuronal development, reduced expression of the histone H3 methyl transferase DOT1L impairs the proliferation and survival of NSCs in the cerebral cortex by upregulating ATF-4 and CHOP expression. Loss of H3 lysine 79 (H3K79) dimethylation in the ATF-4 and CHOP promoters derepresses their expression [141] thereby causing irreversible activation of CHOP-mediated cell death program in NSCs. These data suggest that unresolved ER stress may become deleterious for NSC survival and self-renewal.

Many of the known neurodegenerative disorders and motor neuron diseases such as Huntington's disease, Parkinson's disease, and amyotrophic lateral sclerosis are associated with expression of aggregation prone mutant proteins. Many of these disorders are manifested in middle-aged or older adults; it is not clear if and how NSC dysfunction contributes to their pathogenesis [154–156]. There are however some neurodegenerative disorders that may stem from inability of NSC and/or neuronal progenitor cells to maintain proteostasis. For example, Cockayne syndrome is a neurodegenerative disorder characterized by impaired RNA polymerase I-dependent transcription that causes defective ribosome biogenesis [157]. Other symptoms of the disease are growth retardation, cataracts, alopecia, and premature death. In Cockayne syndrome, reduced synthesis of ribosomal RNAs is associated with overall reduction in the translation efficiency and reduced fidelity of translation. This in turn increases generation of reactive oxygen species, unfolded proteins, CHOP expression, and apoptosis [157]. Another hallmark of the Cockayne syndrome is reduced self-renewal of neuronal progenitors [158].

Spinal cord injury and repeated exposure to pyrethroid insecticides such as deltamethrin causes cognitive decline in adults. Molecular analysis indicates that in both cases cognitive decline is associated with reduced proliferation and increased apoptosis of stem and progenitor cells in the dentate gyrus of the hippocampus indicating that these two disorders may be triggered by reduced neurogenesis [159]. At the molecular level, both disorders are associated with neuronal ER stress and activation of the IERSR. While it is not clear if the IERSR plays a causative role in the development of cognitive decline, it is clear that inability of neuronal precursors to prevent accumulation of unfolded proteins results in the reduced neurogenesis and increased apoptosis which accounts for the development of the cognitive decline. The IERSR also plays important roles in the development and survival of astrocytes and oligodendrocytes [160]. While the IERSR is activated in demyelination disorders, its exact role remains to be elucidated [161–165].

Hormesis, the adaptive preconditioning by induction of mild ER stress, is an interesting concept advanced in the recent years. Adaptive preconditioning to ER stress likely improves proteostasis thereby protecting neurons in some models of neurodegenerative disorders [166]. One potential molecular pathway that facilitates hormesis could be the phosphorylation and activation of Nrf2 by PERK. Nrf2 turns on the expression of antioxidant genes which may reduce free radical-induced damage to macromolecules including proteins and thus protect neuronal progenitors and differentiated neurons [167]. Taken together, it appears that proteostasis plays critical roles in NSC maintenance, self-renewal, differentiation and survival of terminally differentiated neurons. Future studies should test the therapeutic potential of proteostasis modulators for treatment of neuronal disorders.

#### 2.4.2. Hematopoietic Stem/Progenitor Cells (HSCs, HPCs)

Terminally differentiated hematopoietic cells have a finite life span and must be continuously replaced. They are also target of many pathogens. The physiologic replacement of hematopoietic cells as well as their accelerated regeneration in response to pathologic insults requires constant self-renewal and differentiation of HSCs and HPCs. The rate of protein synthesis in HSCs is lower than that of HPCs, likely owing to HSCs' quiescence. Either overstimulation or suppression of protein synthesis impairs HSC function [168]. For example, deletion of Runx1 gene, a transcription factor important for ribosome biogenesis, reduces HSC's ability to proliferate and/or differentiate into downstream progenitors [169]. Importantly, change in the rate of protein synthesis underlies many hematopoietic disorders, collectively known as ribosomopathies. In all known cases of ribosomopathies, a haploinsufficiency of an rRNA biogenesis gene, ribosomal protein gene, or ribosome assembly factor gene reduces the overall number of mature ribosomes (reviewed by [170]). In Diamond-Blackfan anemia (DBA), which is caused by haploinsufficiency of various ribosomal proteins, HSCs give rise to all but the myelogenous lineage [171]. While pathogenesis of ribosomopathies has previously been attributed to imbalance in the ribosomal proteins or ribosomal subunits and subsequent activation of p53-mediated apoptosis by free ribosomal proteins [170], this view has now been convincingly challenged. Specifically, in DBA patients' HSCs, ribosome constitution and relative levels of ribosomal proteins remain constant, but number of ribosomes is reduced. This reduction is associated with preferential reduction in the translation efficiency of a subset of mRNAs with simpler and shorter 5′-UTR, usually associated with efficient translation, and mRNAs whose 5′-UTR contain various motifs including 5′terminal oligopyrymidine tract (5′TOP). The inefficient translation of these mRNAs is attributed to the reduced expression of nonribosomal protein GATA1 [171, 172]. Suppression of GATA1 is sufficient to recapitulate all hallmarks of DBA including repression of HSC differentiation into myelogenous lineage while GATA1's forced expression is sufficient to restore differentiation of HSC into all hematopoietic lineages [171]. These data demonstrate the role of translation in the differentiation of HSC into downstream lineages.

BiP plays an important role in the survival and proliferation of HSCs in the hypoxic niches [173, 174]. Under experimental conditions, HSCs activate the IERSR, particularly PERK/eIF2*α* phosphorylation to a higher extent than the downstream progenitor cells upon treatment with ER stress-inducer tunicamycin. PERK/eIF2*α* phosphorylation arm of the IERSR upregulates CHOP and GADD34 in HSCs and causes selective apoptosis. GADD34, the eIF2*α* phosphatase, can cause eIF2*α* dephosphorylation before ER stress is ameliorated, which is thought to cause oxidative stress and thereby cell death. Consistently, inhibiting activity of this phosphatase by chemical agents protects HSCs from tunicamycin-induced toxicity [175]. GADD34 is also activated upon engraftment of HSCs to bone marrow, and this is associated with low frequency of engraftment [175].

Overexpression of the ER-chaperone ERDJ4 increases the protein-folding capacity of the ER thus reducing the eIF2*α* phosphorylation and GADD34 expression [176, 177]. HSCs overexpressing ERDJ4 engraft into host bone marrow with much higher efficiency due to a low rate of apoptosis attributable to suppression of GADD34 [173]. These data indicate that preventing accumulation of unfolded or misfolded proteins in the ER is critically important for the survival HSCs and their protection from GADD34-induced oxidative stress during engraftment. Inducing low level but sustained eIF2*α* phosphorylation using one of several small chemical inducers of eIF2*α* kinases or eIF2*α* phosphatase inhibitors should be considered for chemical protection of transplanted HSCs [59, 178–181]. Recent reports indicate that estrogen treatment increases the capacity of HSCs to regenerate the hematopoietic system upon transplantation and accelerates regeneration after irradiation by activating the Ire1*α*-Xbp1 pathway [182]. These data are consistent with the notion that transplanted HSCs are under proteotoxic stress and that the Ire1/Xbp1 signaling protects cells from such insults.

As in pluripotent stem cells, the improperly folded proteins in HSCs are degraded by the coordinated action of UPS and autophagy. Autophagy is important for maintaining HSC self-renewal [183] as its inhibition in the HSC compartment leads to defects in stem cell activity resembling those observed in aging and certain disease conditions. These include increased ROS levels, accumulation of damaged protein and organelles, and limited repopulation capacity resulting in multilineage cytopenias [184, 185]. Elevation of ROS levels forces HSCs to choose between elimination by apoptosis or activation [186]. HSCs activate FoxO3-dependent autophagy to avoid apoptosis [187]. Consistently, genetic and chemical interference with autophagy compromises fetal and adult hematopoiesis [184, 186]. In the context of HSCs, UPS also regulates abundance of transcriptional regulators important for HSCs' maintenance and homeostasis such as Notch, c-Myc, c-Kit, and Stat5 [188, 189]. Abundance of other transcription factors required for HSCs' function, including c-Rel, RelA, and GATA2 is also tightly regulated by the IERSR [190].

One strategy HSCs utilize to maintain a pristine proteome is to limit free radical (ROS) damage to existing proteome. HSCs express a protein complex comprising the mitochondrial heat-shock protein mortalin and Dj-1, a direct ROS scavenger to limit damage from oxidative stress that usually emanates from aerobic respiration [191]. Blockade of these protective pathways limits the ability of activated HSCs to return to quiescence, leading to impairments in long-term reconstitution capacity [191–193]. HIF2*α* similarly plays an important role in the survival and engraftment of human HSCs by reducing oxidative stress [193]. Finally, control of mitochondrial translation and energy metabolism play essential roles in homeostasis and mitochondrial protein quality control in HSCs [194].

In summary, HSCs, at variance with their committed progenitors, exhibit decreased translation and increased proteostatic quality control to efficiently preserve stem cell fitness, resolve and overcome pathologic insults, and ensure their proper function and propagation. Chemical enhancers of these cellular processes may have clinical applications in the treatment of ribosomopathies and enhancement of bone marrow grafting upon transplantation.

#### 2.4.3. Mesenchymal Stem Cells (MSCs)

Mesenchymal stem cells are derived from paraxial mesoderm. Differentiation of pluripotent stem cells into mesoderm is mediated by the transcriptional program under the control of ATF-6 and requires expansion of the ER [80]. MSCs give rise to many cell types including osteoblasts, chondrocytes, adipocytes, fibroblasts, and cardiomyocytes [195]. The IERSR signaling drives MSC differentiation into various cell types. Bone morphogenetic protein 2 (BMP2) activates Ire1/XBP-1 signaling and induces BiP expression during chondrocyte differentiation [196]. Both XBP-1 and ATF-6 play essential roles in chondrocyte hypertrophy and differentiation and stimulate endochondral bone formation [197, 198]. Ire1 appears to oppose BMP2-induced osteoblast differentiation [197]. The cysteine-rich ER stress-inducible factor with EGF-like domains 2 is an important mediator of BMP9-regulated osteogenic differentiation of MSCs [199]. Experimentally, arsenic trioxide induces ER stress and reduces the viability of human umbilical cord and bone marrow-derived MSCs while lysophosphatidic acid decreases ER stress-mediated apoptosis triggered by hypoxia and serum deprivation [200]. Knocking down BiP expression activates ER stress-specific caspase cascade in developing chondrocytes [198]. Osteoblast proliferation and differentiation are also impaired in Arl6ip5 knockdown and deficient osteoblasts. Arl6ip5 deficiency enhances ER stress-induced CHOP-mediated apoptosis [201]. Pathologic exposure of MSC in bone marrow to alcohol inhibits osteogenesis while inducing adipogenesis; both associated with ER stress and activation of TNF-*α* signaling. Knocking down ATF-4, CHOP, or TNF-*α* expression reverses ethanol-induced adipogenesis [202]. While these studies indicate that the ER stress signaling can induce apoptosis in MSCs under certain conditions, the role ofthe IERSR in normal biology of these cells remains to be determined.

MSC transplantation has been proposed as a potential therapy for hypoxia/reoxygenation (H/R) damage, i.e., following myocardial infarction. While these cells appear to be highly amenable to transplantation, most will not survive when transplanted into infarcted sites due to very high level of mitochondria-generated ROS which damages cellular proteins [195]. This results in the accumulation of unfolded proteins particularly in the ER and sustained activation of the IERSR which is thought to cause apoptotic death of transplanted cells. Suppression of ROS production under H/R-like conditions (i.e., by delta opioid peptide-DADLE) prevented cell death and reduced the activity of the IERSR regulator PERK Ire1 ATF-6 and BiP to basal level [203]. Currently, nonsurvival of the transplanted stem-progenitor cells into myocardial infarct site is the biggest impediment to cellular therapy. Multiple animal studies have shown that stem and progenitor cells' engraftment into infarcted heart tissue is minimal most likely because the transplanted cells do not survive [204, 205].

Adult multipotent MSC-like cells that highly express CD44 were detected within the vessel wall, particularly within the vascular adventitia. These cells displayed the capacity to deliver vascular smooth muscle cells and contribute to new vessel formation [206]. CD44 expression is reportedly regulated by autophagy flux [207]. Recently, spontaneously beating cardiomyocyte-like cells were generated using vascular adventitia-derived FLK1(+)CD34(+)Sca-1(-) stem cells [208]. MSC-like cell colonies containing these beating cells also expressed high level of CD44(+). MSCs reportedly modulate autophagy flux by releasing exosomes containing miR-125b-5p that resulted in improved cardiac function [209]. These data suggest that transplanted MSC may utilize proteostatic pathways to induce regeneration of host tissue in a paracrine manner. It remains to be seen whether preventing activation of the IERSR signaling or low-level preemptive and/or selective activation of the IERSR signaling pathways or autophagy will enhance the survival of MSCs transplanted into myocardial infarct site. Numerous inhibitors and activators of ER stress response and autophagy developed in recent years can be used to answer these questions [59, 178, 179, 210–214].

#### 2.4.4. Multilineage Stress Enduring (MUSE) Stem Cells

Recently, a population of stem cell-like cells isolated from adipose tissue was shown to be highly resistant to ER stress compared to other mesenchymal or adipose stem cells. These lipoaspirated MUSE cells express high levels of Oct-4, Sox-2, Klf4, and c-Myc and are capable of differentiating into endodermal, mesodermal, and ectodermal lineages suggesting that they may be stem cells [215]. Nevertheless, further characterization of MUSE cells is needed for establishing their credentials as *bona fide* pluripotent stem cells. Cells of similar properties have been isolated from other tissues and shown to have the ability to home to the site of ischemia-reperfusion (I/R) injury in the lungs, heart, brain, osteochondral defect created in rat patellar grove, carbon tetrachloride-induced liver injury, intracerebral hemorrhage, or kidney damage induced by Adriamycin [216–222]. In all these cases, MUSE cells differentiated into and restored function to injured tissue and organ. These data indicate that MUSE cells may be highly amenable to transplantation for tissue repair under pathological conditions. Currently, little is known about the molecular basis of stress resistance in MUSE cells. This knowledge would be invaluable for the identification and development of chemical agents that would help improve the transplantation efficiency of all stem cells.

#### 2.4.5. Cardiac Stem Progenitor Cells (CSPCs)

Cardiac stem cells, which possess the capacity of self-renewal, likely descend from various lineages that contribute to formation of the adult heart. Stem and progenitor cells with different immunophenotype possess cardioregenerative potential and improve cardiac function when transplanted into the heart after myocardial infarction (see review [205]). Interestingly this was not associated with a considerable myocardial regeneration. CSPCs reportedly express c-kit, Sca-1, PDGFR*α*, and Isl1 [223–225]. Interaction of Notch receptor with its ligand Jagged has a profound effect on the activation of CSPC. Jagged/Notch signaling induces CSPCs to enter a replicative state resulting in the generation of transient-amplifying cells in proliferative state [226, 227].

Cardiac differential PARM-1 (prostatic androgen repressed message-1) is an ER-resident protein and appears to play an important role in cardiomyogenic differentiation by impinging on BMP/SMAD signaling [228]. Deletion of the ER chaperone ERp44 causes ER and mitochondrial stress in CSCs and results in apoptotic cell death [228]. Mutations in the ER chaperone calreticulin cause neonatal death by interfering with normal heart development [229, 230]. Another chaperone, BAG-3, interacts with both the heat-shock family of chaperones, ribosomal proteins, and its own client proteins. Haploinsufficiency of BAG-3 causes heart failure which is significantly exacerbated by proteasome inhibitors [231]. These data indicate that the IERSR plays an important role in the survival and differentiation of CSCs not only *in vitro* but also upon their engraftment into the site of myocardial infarction. Whether pharmacologic modifiers of the IERSR can have beneficial effects on CSC survival, self-renewal, and differentiation into mature cardiomyocytes remains to be determined.

#### 2.4.6. Skeletal Muscle Stem Cells (SMSCs)

Adult skeletal muscle is a stable tissue under normal conditions, but it has a remarkable ability to regenerate by virtue of its resident stem cells. The SMSCs exist in a quiescent state with a very low turnover [232]. However, in response to an activating signal, SMSCs leave the quiescent state and enter the cell cycle [233]. While a small proportion of these newly proliferated SMSCs exit cell cycle early on and return to the quiescent state, a much larger proportion will proliferate for several generations. This expanded population of SMSCs will undergo cellular differentiation, fusion, growth, and maturation to form myofibers [234, 235]. Maintenance of SMSCs in quiescent state requires basal surveillance mechanisms to preserve the quality of the proteome and maintain cellular homoeostasis [234, 236]. Tight control of translation and the IERSR pathways preserves the quiescent state. Specifically, translation of Dek protein, which promotes SMSCs proliferative expansion through a mechanism involving miR-489-dependent process, is actively suppressed [236]. Selective repression of translation, mediated by eIF2*α* phosphorylation, is also required for the maintenance of SMSCs quiescence [237, 238]. Consistently, SMSCs carrying a nonphosphorylatable eIF2*α* allele or deficient in PERK spontaneously undergo myogenic differentiation losing the capacity to self-renew (Zismanov et al., [238]). SMSCs sequester Myf5 mRNA into RNA granules and swiftly translate it once cells reinitiate the proliferative and myogenic differentiation [237]. Autophagy plays an essential role in SMSC quiescence as well as response to muscle injury. Autophagy is maintained at low constitutive level in quiescent SMSCs to remove damaged or unneeded organelles and proteins [239, 240]. Upon muscle injury, autophagy is activated to provide energy as well as establish proteome and organelle composition suitable for the SMSC proliferation, differentiation, and myotube formation [239, 241–243]. Deletion of Atg7 in SMSCs at a young age causes accumulation of damaged mitochondria, which increases ROS levels, damaging proteins and DNA and thereby causing senescence of SMSCs [240]. Pharmacological inhibition of ROS in Atg7-deficient SMSCs prevents senescence and restores self-renewal capacity providing further evidence that maintaining a pristine proteome is essential for SMSCs survival, proliferation, and differentiation [240, 244, 245].

#### 2.4.7. Intestinal Stem Cells (ISCs)

Intestinal epithelium is characterized by constant proliferation of cells in the base of crypt and shedding of cells at the top of the villi. To keep up with the demand for high turnover using a relatively small stem cell pool, ISCs are organized in a hierarchical manner [246]. The crypt base columnar stem cells (CBSCs) are fairly abundant and actively proliferate; they are characterized by high Wnt signaling [247, 248]. CBSCs give rise to proliferative cells variously known as transiently amplifying or long-term amplifying cells that has finite proliferative potential and differentiate into various cells in the crypt. Reserve ISC cycle slower; these cells are resistant to DNA damage-inducing agents and negative for Wnt signaling [249]. The ER chaperone BiP, Xbp-1S, and phosphorylated eIF2*α* are absent in the CBSCs but highly expressed in transiently amplifying cells and other differentiated cells in the crypts [249]. Xbp-1 plays a critical role in ISC homeostasis [250, 251]. Induction of ER stress by chemical agents or through deletion of BiP causes a loss of CBSC through differentiation and dismantling of the crypt. These effects appear to be totally dependent on eIF2*α* phosphorylation but only partially on PERK [249]. Another eIF2*α* kinase, PKR, may also be involved in this process [252]. Interestingly, while deletion of BiP specifically in CBSCs causes their loss due to accelerated differentiation, the CBSC population is restored in the following days indicating that reserve ISCs repopulate the crypt [249].

## 3. Concluding Remarks and Future Directions

From the time of fertilization to death, development and survival of complex multicellular organism are dependent on communicating with and responding to extracellular environment. In the case of mammalian development, perhaps there is no better demonstration of this than embryonic diapause when blastocysts suspend normal embryonic development program and resume it when conditions become more conducive to normal development [253, 254]. Complex multicellular organisms must also preserve a reservoir of stem and progenitor cells in immaculate condition over its entire lifespan to maintain their organ and tissues and respond to pathologic insults. Accumulation of incorrectly produced or processed proteins in stem and progenitor cells could have dire consequences for tissue homeostasis and the health of the organism. Consequently, a multitude of interconnected and coordinated mechanisms has evolved that allow these cells to maintain a pristine proteome.

Understanding how stem and progenitor cells maintain proteostasis would help us develop strategies to prevent and/or treat proteostatic disorders and improve our ability to explore the therapeutic potential of stem and progenitor cells by providing them with appropriate culture conditions when these cells are expanded *in vitro* as well as modifying the *in vivo* environment when they are transplanted. In this review, we recapitulated our current knowledge of the processes employed by various stem and progenitor cells to maintain a pristine proteome and how disturbances of these processes affect human health. Although our insight into proteostasis has tremendously advanced in the last decade, there are still many areas where more progress is needed. For example, it is unclear why defects in apparently universal mechanisms for proteostasis can affect the organism at unpredictable stages of development. A second important question that needs to be answered is how the various networks or mechanisms involved in mRNA translation, protein folding, inhibition of aggregation, and removal of faulty proteins are coordinated in stem cells.

## References

[1] Treaster S. B., Ridgway I. D., Richardson C. A., Gaspar M. B., Chaudhuri A. R., Austad S. N. (2014). Superior proteome stability in the longest lived animal. *Age*.

[2] Noormohammadi A., Calculli G., Gutierrez-Garcia R., Khodakarami A., Koyuncu S., Vilchez D. (2018). Mechanisms of protein homeostasis (proteostasis) maintain stem cell identity in mammalian pluripotent stem cells. *Cellular and Molecular Life Sciences*.

[3] You K. T., Park J., Kim V. N. (2015). Role of the small subunit processome in the maintenance of pluripotent stem cells. *Genes & Development*.

[4] Tafforeau L., Zorbas C., Langhendries J. L. (2013). The complexity of human ribosome biogenesis revealed by systematic nucleolar screening of pre-rRNA processing factors. *Molecular Cell*.

[5] Kratochvílová K., Moráň L., Paďourová S. (2016). The role of the endoplasmic reticulum stress in stemness, pluripotency and development. *European Journal of Cell Biology*.

[6] Inaba M., Yamashita Y. M. (2012). Asymmetric stem cell division: precision for robustness. *Cell Stem Cell*.

[7] Rujano M. A., Bosveld F., Salomons F. A. (2006). Polarised asymmetric inheritance of accumulated protein damage in higher eukaryotes. *PLoS Biology*.

[8] Ogrodnik M., Salmonowicz H., Brown R. (2014). Dynamic JUNQ inclusion bodies are asymmetrically inherited in mammalian cell lines through the asymmetric partitioning of vimentin. *Proceedings of the National Academy of Sciences of the United States of America*.

[9] Toyama B. H., Savas J. N., Park S. K. (2013). Identification of long-lived proteins reveals exceptional stability of essential cellular structures. *Cell*.

[10] Garcia-Prat L., Sousa-Victor P., Munoz-Canoves P. (2017). Proteostatic and metabolic control of stemness. *Cell Stem Cell*.

[11] Lee H. J., Gutierrez-Garcia R., Vilchez D. (2017). Embryonic stem cells: a novel paradigm to study proteostasis?. *The FEBS Journal*.

[12] Knobloch M., Braun S. M., Zurkirchen L. (2013). Metabolic control of adult neural stem cell activity by Fasn-dependent lipogenesis. *Nature*.

[13] Bradley E., Bieberich E., Mivechi N. F., Tangpisuthipongsa D., Wang G. (2012). Regulation of embryonic stem cell pluripotency by heat shock protein 90. *Stem Cells*.

[14] Kramer G., Boehringer D., Ban N., Bukau B. (2009). The ribosome as a platform for co-translational processing, folding and targeting of newly synthesized proteins. *Nature Structural & Molecular Biology*.

[15] Schieweck R., Popper B., Kiebler M. A. (2016). Co-translational folding: a novel modulator of local protein expression in mammalian neurons?. *Trends in Genetics*.

[16] Albanèse V., Yam A. Y. W., Baughman J., Parnot C., Frydman J. (2006). Systems analyses reveal two chaperone networks with distinct functions in eukaryotic cells. *Cell*.

[17] Reid D. W., Nicchitta C. V. (2012). Primary role for endoplasmic reticulum-bound ribosomes in cellular translation identified by ribosome profiling. *The Journal of Biological Chemistry*.

[18] Balchin D., Hayer-Hartl M., Hartl F. U. (2016). In vivo aspects of protein folding and quality control. *Science*.

[19] Reithinger J. H., Kim J. E. H., Kim H. (2013). Sec62 protein mediates membrane insertion and orientation of moderately hydrophobic signal anchor proteins in the endoplasmic reticulum (ER). *Journal of Biological Chemistry*.

[20] Vilchez D., Boyer L., Morantte I. (2012). Increased proteasome activity in human embryonic stem cells is regulated by PSMD11. *Nature*.

[21] Strikoudis A., Guillamot M., Aifantis I. (2014). Regulation of stem cell function by protein ubiquitylation. *EMBO Reports*.

[22] Liu K., Zhao Q., Liu P. (2016). ATG3-dependent autophagy mediates mitochondrial homeostasis in pluripotency acquirement and maintenance. *Autophagy*.

[23] Pan H., Cai N., Li M., Liu G. H., Izpisua Belmonte J. C. (2013). Autophagic control of cell ‘stemness’. *EMBO Molecular Medicine*.

[24] Brehme M., Voisine C., Rolland T. (2014). A chaperome subnetwork safeguards proteostasis in aging and neurodegenerative disease. *Cell Reports*.

[25] McClellan A. J., Xia Y., Deutschbauer A. M., Davis R. W., Gerstein M., Frydman J. (2007). Diverse cellular functions of the Hsp90 molecular chaperone uncovered using systems approaches. *Cell*.

[26] Labbadia J., Morimoto R. I. (2015). The biology of proteostasis in aging and disease. *Annual Review of Biochemistry*.

[27] Cabrita L. D., Cassaignau A. M. E., Launay H. M. M. (2016). A structural ensemble of a ribosome-nascent chain complex during cotranslational protein folding. *Nature Structural & Molecular Biology*.

[28] Cuéllar J., Martín-Benito J., Scheres S. H. W. (2008). The structure of CCT–Hsc70_NBD_ suggests a mechanism for Hsp70 delivery of substrates to the chaperonin. *Nature Structural & Molecular Biology*.

[29] Morgner N., Schmidt C., Beilsten-Edmands V. (2015). Hsp70 forms antiparallel dimers stabilized by post-translational modifications to position clients for transfer to Hsp90. *Cell Reports*.

[30] Kalisman N., Adams C. M., Levitt M. (2012). Subunit order of eukaryotic TRiC/CCT chaperonin by cross-linking, mass spectrometry, and combinatorial homology modeling. *Proceedings of the National Academy of Sciences of the United States of America*.

[31] Taipale M., Krykbaeva I., Koeva M. (2012). Quantitative analysis of HSP90-client interactions reveals principles of substrate recognition. *Cell*.

[32] Son Y. S., Park J. H., Kang Y. K. (2005). Heat shock 70-kDa protein 8 isoform 1 is expressed on the surface of human embryonic stem cells and downregulated upon differentiation. *Stem Cells*.

[33] Prinsloo E., Setati M. M., Longshaw V. M., Blatch G. L. (2009). Chaperoning stem cells: a role for heat shock proteins in the modulation of stem cell self-renewal and differentiation?. *BioEssays*.

[34] Saretzki G., Armstrong L., Leake A., Lako M., von Zglinicki T. (2004). Stress defense in murine embryonic stem cells is superior to that of various differentiated murine cells. *Stem Cells*.

[35] Saretzki G., Walter T., Atkinson S. (2008). Downregulation of multiple stress defense mechanisms during differentiation of human embryonic stem cells. *Stem Cells*.

[36] Noormohammadi A., Khodakarami A., Gutierrez-Garcia R. (2016). Somatic increase of CCT8 mimics proteostasis of human pluripotent stem cells and extends *C. elegans* lifespan. *Nature Communications*.

[37] Son J. S., Jeong Y. C., Kwon Y. B. (2015). Regulatory effect of bee venom on methamphetamine-induced cellular activities in prefrontal cortex and nucleus accumbens in mice. *Biological & Pharmaceutical Bulletin*.

[38] Ron D., Harding H. P. (2012). Protein-folding homeostasis in the endoplasmic reticulum and nutritional regulation. *Cold Spring Harbor Perspectives in Biology*.

[39] Malhotra J. D., Kaufman R. J. (2007). The endoplasmic reticulum and the unfolded protein response. *Seminars in Cell & Developmental Biology*.

[40] Ron D., Walter P. (2007). Signal integration in the endoplasmic reticulum unfolded protein response. *Nature Reviews Molecular Cell Biology*.

[41] Milner R. E., Famulski K. S., Michalak M. (1992). Calcium binding proteins in the sarcoplasmic/endoplasmic reticulum of muscle and nonmuscle cells. *Molecular and Cellular Biochemistry*.

[42] Ron D. (2004). Signaling cascades regulating NMDA receptor sensitivity to ethanol. *The Neuroscientist*.

[43] Ron D., Oyadomari S. (2004). Lipid phase perturbations and the unfolded protein response. *Developmental Cell*.

[44] Oyadomari S., Yun C., Fisher E. A. (2006). Cotranslocational degradation protects the stressed endoplasmic reticulum from protein overload. *Cell*.

[45] Kadowaki H., Nagai A., Maruyama T. (2015). Pre-emptive quality control protects the ER from protein overload via the proximity of ERAD components and SRP. *Cell Reports*.

[46] Yewdell J. W. (2005). Serendipity strikes twice: the discovery and rediscovery of defective ribosomal products (DRiPS). *Cellular and Molecular Biology (Noisy-le-Grand, France)*.

[47] Schubert U., Antón L. C., Gibbs J., Norbury C. C., Yewdell J. W., Bennink J. R. (2000). Rapid degradation of a large fraction of newly synthesized proteins by proteasomes. *Nature*.

[48] Shuda M., Kondoh N., Imazeki N. (2003). Activation of the ATF6, XBP1 and grp78 genes in human hepatocellular carcinoma: a possible involvement of the ER stress pathway in hepatocarcinogenesis. *Journal of Hepatology*.

[49] Okada T., Yoshida H., Akazawa R., Negishi M., Mori K. (2002). Distinct roles of activating transcription factor 6 (ATF6) and double-stranded RNA-activated protein kinase-like endoplasmic reticulum kinase (PERK) in transcription during the mammalian unfolded protein response. *The Biochemical Journal*.

[50] Schuldt A. (2014). Protein metabolism: a channel for ERAD. *Nature Reviews Molecular Cell Biology*.

[51] Ninagawa S., Okada T., Sumitomo Y. (2015). Forcible destruction of severely misfolded mammalian glycoproteins by the non-glycoprotein ERAD pathway. *The Journal of Cell Biology*.

[52] Pan S., Cheng X., Sifers R. N. (2013). Golgi-situated endoplasmic reticulum *α*-1, 2-mannosidase contributes to the retrieval of ERAD substrates through a direct interaction with *γ*-COP. *Molecular Biology of the Cell*.

[53] Sato N., Urano F., Yoon Leem J. (2000). Upregulation of BiP and CHOP by the unfolded-protein response is independent of presenilin expression. *Nature Cell Biology*.

[54] Vattem K. M., Wek R. C. (2004). Reinitiation involving upstream ORFs regulates ATF4 mRNA translation in mammalian cells. *Proceedings of the National Academy of Sciences of the United States of America*.

[55] Bernasconi R., Molinari M. (2011). ERAD and ERAD tuning: disposal of cargo and of ERAD regulators from the mammalian ER. *Current Opinion in Cell Biology*.

[56] Cullinan S. B., Diehl J. A. (2004). PERK-dependent activation of Nrf2 contributes to redox homeostasis and cell survival following endoplasmic reticulum stress. *The Journal of Biological Chemistry*.

[57] Cullinan S. B., Zhang D., Hannink M., Arvisais E., Kaufman R. J., Diehl J. A. (2003). Nrf2 is a direct PERK substrate and effector of PERK-dependent cell survival. *Molecular and Cellular Biology*.

[58] Deng J., Lu P. D., Zhang Y. (2004). Translational repression mediates activation of nuclear factor kappa B by phosphorylated translation initiation factor 2. *Molecular and Cellular Biology*.

[59] Bai H., Chen T., Ming J. (2013). Dual activators of protein kinase R (PKR) and protein kinase R-like kinase PERK identify common and divergent catalytic targets. *ChemBioChem*.

[60] Philippe C., Dubrac A., Quelen C. (2016). PERK mediates the IRES-dependent translational activation of mRNAs encoding angiogenic growth factors after ischemic stress. *Science Signaling*.

[61] Wang X., Wang P., Zhu Y., Zhang Z., Zhang J., Wang H. (2016). MicroRNA-520a attenuates proliferation of Raji cells through inhibition of AKT1/NF-*κ*B and PERK/eIF2*α* signaling pathway. *Oncology Reports*.

[62] Gupta S., Read D. E., Deepti A. (2012). Perk-dependent repression of miR-106b-25 cluster is required for ER stress-induced apoptosis. *Cell Death & Disease*.

[63] Gupta S., Giricz Z., Natoni A. (2012). NOXA contributes to the sensitivity of PERK-deficient cells to ER stress. *FEBS Letters*.

[64] Bobrovnikova-Marjon E., Pytel D., Riese M. J. (2012). PERK utilizes intrinsic lipid kinase activity to generate phosphatidic acid, mediate Akt activation, and promote adipocyte differentiation. *Molecular and Cellular Biology*.

[65] Bobrovnikova-Marjon E., Hatzivassiliou G., Grigoriadou C. (2008). PERK-dependent regulation of lipogenesis during mouse mammary gland development and adipocyte differentiation. *Proceedings of the National Academy of Sciences of the United States of America*.

[66] Harding H. P., Zeng H., Zhang Y. (2001). Diabetes mellitus and exocrine pancreatic dysfunction in perk-/- mice reveals a role for translational control in secretory cell survival. *Molecular Cell*.

[67] Julier C., Nicolino M. (2010). Wolcott-Rallison syndrome. *Orphanet Journal of Rare Diseases*.

[68] Senee V., Vattem K. M., Delepine M. (2004). Wolcott-Rallison syndrome: clinical, genetic, and functional study of EIF2AK3 mutations and suggestion of genetic heterogeneity. *Diabetes*.

[69] Calfon M., Zeng H., Urano F. (2002). IRE1 couples endoplasmic reticulum load to secretory capacity by processing the XBP-1 mRNA. *Nature*.

[70] Takayanagi S., Fukuda R., Takeuchi Y., Tsukada S., Yoshida K. (2013). Gene regulatory network of unfolded protein response genes in endoplasmic reticulum stress. *Cell Stress & Chaperones*.

[71] Hollien J., Lin J. H., Li H., Stevens N., Walter P., Weissman J. S. (2009). Regulated Ire1-dependent decay of messenger RNAs in mammalian cells. *The Journal of Cell Biology*.

[72] Hollien J., Weissman J. S. (2006). Decay of endoplasmic reticulum-localized mRNAs during the unfolded protein response. *Science*.

[73] Moore K. A., Hollien J. (2012). The unfolded protein response in secretory cell function. *Annual Review of Genetics*.

[74] Nishitoh H., Matsuzawa A., Tobiume K. (2002). ASK1 is essential for endoplasmic reticulum stress-induced neuronal cell death triggered by expanded polyglutamine repeats. *Genes & Development*.

[75] Reimold A. M., Etkin A., Clauss I. (2000). An essential role in liver development for transcription factor XBP-1. *Genes & Development*.

[76] Sano R., Hou Y. C. C., Hedvat M. (2012). Endoplasmic reticulum protein BI-1 regulates Ca^2+^-mediated bioenergetics to promote autophagy. *Genes & Development*.

[77] Sano R., Reed J. C. (2013). ER stress-induced cell death mechanisms. *Biochimica et Biophysica Acta*.

[78] Kober L., Zehe C., Bode J. (2012). Development of a novel ER stress based selection system for the isolation of highly productive clones. *Biotechnology and Bioengineering*.

[79] Yoshida H., Nadanaka S., Sato R., Mori K. (2006). XBP1 is critical to protect cells from endoplasmic reticulum stress: evidence from site-2 protease-deficient Chinese hamster ovary cells. *Cell Structure and Function*.

[80] Kroeger H., Grimsey N., Paxman R. (2018). The unfolded protein response regulator ATF6 promotes mesodermal differentiation. *Science Signaling*.

[81] Yamamoto K., Sato T., Matsui T. (2007). Transcriptional induction of mammalian ER quality control proteins is mediated by single or combined action of ATF6alpha and XBP1. *Developmental Cell*.

[82] Luo S., Mao C., Lee B., Lee A. S. (2006). GRP78/BiP is required for cell proliferation and protecting the inner cell mass from apoptosis during early mouse embryonic development. *Molecular and Cellular Biology*.

[83] Verfaillie T., Rubio N., Garg A. D. (2012). PERK is required at the ER-mitochondrial contact sites to convey apoptosis after ROS-based ER stress. *Cell Death and Differentiation*.

[84] Senft D., Ronai Z.’e. A. (2015). UPR, autophagy, and mitochondria crosstalk underlies the ER stress response. *Trends in Biochemical Sciences*.

[85] Wang J., Lee J., Liem D., Ping P. (2017). HSPA5 gene encoding Hsp70 chaperone BiP in the endoplasmic reticulum. *Gene*.

[86] Mao C., Wang M., Luo B. (2010). Targeted mutation of the mouse Grp94 gene disrupts development and perturbs endoplasmic reticulum stress signaling. *PLoS One*.

[87] Wanderling S., Simen B. B., Ostrovsky O. (2007). GRP94 is essential for mesoderm induction and muscle development because it regulates insulin-like growth factor secretion. *Molecular Biology of the Cell*.

[88] Xu H., Tsang K. S., Wang Y., Chan J. C. N., Xu G., Gao W. Q. (2014). Unfolded protein response is required for the definitive endodermal specification of mouse embryonic stem cells via Smad2 and *β*-catenin signaling. *The Journal of Biological Chemistry*.

[89] Vilchez D., Simic M. S., Dillin A. (2014). Proteostasis and aging of stem cells. *Trends in Cell Biology*.

[90] Hochstrasser M. (1996). Ubiquitin-dependent protein degradation. *Annual Review of Genetics*.

[91] Pickart C. M. (2001). Mechanisms underlying ubiquitination. *Annual Review of Biochemistry*.

[92] Werner A., Manford A. G., Rape M. (2017). Ubiquitin-dependent regulation of stem cell biology. *Trends in Cell Biology*.

[93] Buckley S. M., Aranda-Orgilles B., Strikoudis A. (2012). Regulation of pluripotency and cellular reprogramming by the ubiquitin-proteasome system. *Cell Stem Cell*.

[94] Finley D. (2009). Recognition and processing of ubiquitin-protein conjugates by the proteasome. *Annual Review of Biochemistry*.

[95] Assou S., Cerecedo D., Tondeur S. (2009). A gene expression signature shared by human mature oocytes and embryonic stem cells. *BMC Genomics*.

[96] Pathare G. R., Nagy I., Bohn S. (2012). The proteasomal subunit Rpn6 is a molecular clamp holding the core and regulatory subcomplexes together. *Proceedings of the National Academy of Sciences of the United States of America*.

[97] Vilchez D., Boyer L., Lutz M. (2013). FOXO4 is necessary for neural differentiation of human embryonic stem cells. *Aging Cell*.

[98] Jang J., Wang Y., Kim H. S., Lalli M. A., Kosik K. S. (2014). Nrf2, a regulator of the proteasome, controls self-renewal and pluripotency in human embryonic stem cells. *Stem Cells*.

[99] Zhao X., Heng J. I. T., Guardavaccaro D. (2008). The HECT-domain ubiquitin ligase Huwe1 controls neural differentiation and proliferation by destabilizing the N-Myc oncoprotein. *Nature Cell Biology*.

[100] Hernebring M., Fredriksson Å., Liljevald M. (2013). Removal of damaged proteins during ES cell fate specification requires the proteasome activator PA28. *Scientific Reports*.

[101] Hernebring M., Brolen G., Aguilaniu H., Semb H., Nystrom T. (2006). Elimination of damaged proteins during differentiation of embryonic stem cells. *Proceedings of the National Academy of Sciences of the United States of America*.

[102] Cuervo A. M. (2004). Autophagy: in sickness and in health. *Trends in Cell Biology*.

[103] Mizushima N., Komatsu M. (2011). Autophagy: renovation of cells and tissues. *Cell*.

[104] Mizushima N., Levine B. (2010). Autophagy in mammalian development and differentiation. *Nature Cell Biology*.

[105] Rodolfo C., Di Bartolomeo S., Cecconi F. (2016). Autophagy in stem and progenitor cells. *Cellular and Molecular Life Sciences*.

[106] Vilchez D., Saez I., Dillin A. (2014). The role of protein clearance mechanisms in organismal ageing and age-related diseases. *Nature Communications*.

[107] Nixon R. A. (2013). The role of autophagy in neurodegenerative disease. *Nature Medicine*.

[108] Labbadia J., Morimoto R. I. (2015). Repression of the heat shock response is a programmed event at the onset of reproduction. *Molecular Cell*.

[109] Martinez-Vicente M., Cuervo A. M. (2007). Autophagy and neurodegeneration: when the cleaning crew goes on strike. *Lancet Neurology*.

[110] Cuervo A. M. (2010). Chaperone-mediated autophagy: selectivity pays off. *Trends in Endocrinology and Metabolism*.

[111] Kincaid M. M., Cooper A. A. (2007). Misfolded proteins traffic from the endoplasmic reticulum (ER) due to ER export signals. *Molecular Biology of the Cell*.

[112] Kincaid M. M., Cooper A. A. (2007). ERADicate ER stress or die trying. *Antioxidants & Redox Signaling*.

[113] Kudo T. (2003). Involvement of unfolded protein responses in neurodegeneration. *Nihon Shinkei Seishin Yakurigaku Zasshi*.

[114] Baldridge R. D., Rapoport T. A. (2016). Autoubiquitination of the Hrd1 ligase triggers protein retrotranslocation in ERAD. *Cell*.

[115] Carvalho P., Stanley A. M., Rapoport T. A. (2010). Retrotranslocation of a misfolded luminal ER protein by the ubiquitin-ligase Hrd1p. *Cell*.

[116] Vembar S. S., Brodsky J. L. (2008). One step at a time: endoplasmic reticulum-associated degradation. *Nature Reviews Molecular Cell Biology*.

[117] Keum Y. S., Owuor E. D., Kim B. R., Hu R., Kong A. N. T. (2003). Involvement of Nrf2 and JNK1 in the activation of antioxidant responsive element (ARE) by chemopreventive agent phenethyl isothiocyanate (PEITC). *Pharmaceutical Research*.

[118] Kwak M. K., Wakabayashi N., Itoh K., Motohashi H., Yamamoto M., Kensler T. W. (2003). Modulation of gene expression by cancer chemopreventive dithiolethiones through the Keap1-Nrf2 pathway. Identification of novel gene clusters for cell survival. *The Journal of Biological Chemistry*.

[119] Kwak M. K., Wakabayashi N., Greenlaw J. L., Yamamoto M., Kensler T. W. (2003). Antioxidants enhance mammalian proteasome expression through the Keap1-Nrf2 signaling pathway. *Molecular and Cellular Biology*.

[120] Pickering A. M., Linder R. A., Zhang H., Forman H. J., Davies K. J. A. (2012). Nrf2-dependent induction of proteasome and Pa28*αβ* regulator are required for adaptation to oxidative stress. *Journal of Biological Chemistry*.

[121] Radhakrishnan S. K., Lee C. S., Young P., Beskow A., Chan J. Y., Deshaies R. J. (2010). Transcription factor Nrf1 mediates the proteasome recovery pathway after proteasome inhibition in mammalian cells. *Molecular Cell*.

[122] Zhang Y., Lucocq J. M., Yamamoto M., Hayes J. D. (2007). The NHB1 (N-terminal homology box 1) sequence in transcription factor Nrf1 is required to anchor it to the endoplasmic reticulum and also to enable its asparagine-glycosylation. *The Biochemical Journal*.

[123] Steffen J., Seeger M., Koch A., Kruger E. (2010). Proteasomal degradation is transcriptionally controlled by TCF11 via an ERAD-dependent feedback loop. *Molecular Cell*.

[124] Baird L., Tsujita T., Kobayashi E. H. (2017). A homeostatic shift facilitates endoplasmic reticulum proteostasis through transcriptional integration of proteostatic stress response pathways. *Molecular and Cellular Biology*.

[125] Wang W., Chan J. Y. (2006). Nrf1 is targeted to the endoplasmic reticulum membrane by an N-terminal transmembrane domain. Inhibition of nuclear translocation and transacting function. *The Journal of Biological Chemistry*.

[126] Ogata M., Hino S. I., Saito A. (2006). Autophagy is activated for cell survival after endoplasmic reticulum stress. *Molecular and Cellular Biology*.

[127] Kaushik S., Cuervo A. M. (2018). The coming of age of chaperone-mediated autophagy. *Nature Reviews Molecular Cell Biology*.

[128] Houck S. A., Ren H. Y., Madden V. J. (2014). Quality control autophagy degrades soluble ERAD-resistant conformers of the misfolded membrane protein GnRHR. *Molecular Cell*.

[129] B’chir W., Maurin A.-C., Carraro V. (2013). The eIF2*α*/ATF4 pathway is essential for stress-induced autophagy gene expression. *Nucleic Acids Research*.

[130] Dunlop E. A., Hunt D. K., Acosta-Jaquez H. A., Fingar D. C., Tee A. R. (2011). ULK1 inhibits mTORC1 signaling, promotes multisite raptor phosphorylation and hinders substrate binding. *Autophagy*.

[131] Kim J., Kundu M., Viollet B., Guan K. L. (2011). AMPK and mTOR regulate autophagy through direct phosphorylation of Ulk1. *Nature Cell Biology*.

[132] Ramming T., Appenzeller-Herzog C. (2013). Destroy and exploit: catalyzed removal of hydroperoxides from the endoplasmic reticulum. *International Journal of Cell Biology*.

[133] Qin L., Wang Z., Tao L., Wang Y. (2010). ER stress negatively regulates AKT/TSC/mTOR pathway to enhance autophagy. *Autophagy*.

[134] Ohoka N., Yoshii S., Hattori T., Onozaki K., Hayashi H. (2005). TRB3, a novel ER stress-inducible gene, is induced via ATF4-CHOP pathway and is involved in cell death. *The EMBO Journal*.

[135] Kouroku Y., Fujita E., Tanida I. (2007). ER stress (PERK/eIF2*α* phosphorylation) mediates the polyglutamine-induced LC3 conversion, an essential step for autophagy formation. *Cell Death and Differentiation*.

[136] Deegan S., Koryga I., Glynn S. A., Gupta S., Gorman A. M., Samali A. (2015). A close connection between the PERK and IRE arms of the UPR and the transcriptional regulation of autophagy. *Biochemical and Biophysical Research Communications*.

[137] Mochida K., Oikawa Y., Kimura Y. (2015). Receptor-mediated selective autophagy degrades the endoplasmic reticulum and the nucleus. *Nature*.

[138] Smith M. D., Harley M. E., Kemp A. J. (2018). CCPG1 is a non-canonical autophagy cargo receptor essential for ER-phagy and pancreatic ER Proteostasis. *Developmental Cell*.

[139] Cho Y. M., Jang Y. S., Jang Y. M. (2009). Induction of unfolded protein response during neuronal induction of rat bone marrow stromal cells and mouse embryonic stem cells. *Experimental & Molecular Medicine*.

[140] Gage F. H. (2000). Mammalian neural stem cells. *Science*.

[141] Roidl D., Hellbach N., Bovio P. P. (2016). DOT1L activity promotes proliferation and protects cortical neural stem cells from activation of ATF4-DDIT3-mediated ER stress in vitro. *Stem Cells*.

[142] Mansouri S., Barde S., Ortsäter H. (2013). GalR3 activation promotes adult neural stem cell survival in response to a diabetic *milieu*. *Journal of Neurochemistry*.

[143] Yazdankhah M., Farioli-Vecchioli S., Tonchev A. B., Stoykova A., Cecconi F. (2014). The autophagy regulators Ambra1 and Beclin 1 are required for adult neurogenesis in the brain subventricular zone. *Cell Death & Disease*.

[144] Laguesse S., Creppe C., Nedialkova D. D. (2015). A dynamic unfolded protein response contributes to the control of cortical neurogenesis. *Developmental Cell*.

[145] Mimura N., Yuasa S., Soma M. (2008). Altered quality control in the endoplasmic reticulum causes cortical dysplasia in knock-in mice expressing a mutant BiP. *Molecular and Cellular Biology*.

[146] Hayashi A., Kasahara T., Iwamoto K. (2007). The role of brain-derived neurotrophic factor (BDNF)-induced XBP1 splicing during brain development. *The Journal of Biological Chemistry*.

[147] Zhang X., Szabo E., Michalak M., Opas M. (2007). Endoplasmic reticulum stress during the embryonic development of the central nervous system in the mouse. *International Journal of Developmental Neuroscience*.

[148] Frank C. L., Ge X., Xie Z., Zhou Y., Tsai L. H. (2010). Control of activating transcription factor 4 (ATF4) persistence by multisite phosphorylation impacts cell cycle progression and neurogenesis. *The Journal of Biological Chemistry*.

[149] Ming G. L., Song H. (2011). Adult neurogenesis in the mammalian brain: significant answers and significant questions. *Neuron*.

[150] Kawada K., Iekumo T., Saito R. (2014). Aberrant neuronal differentiation and inhibition of dendrite outgrowth resulting from endoplasmic reticulum stress. *Journal of Neuroscience Research*.

[151] Kikkert M., Doolman R., Dai M. (2004). Human HRD1 is an E3 ubiquitin ligase involved in degradation of proteins from the endoplasmic reticulum. *The Journal of Biological Chemistry*.

[152] Kaneko M., Ishiguro M., Niinuma Y., Uesugi M., Nomura Y. (2002). Human HRD1 protects against ER stress-induced apoptosis through ER-associated degradation. *FEBS Letters*.

[153] Bertolotti A., Zhang Y., Hendershot L. M., Harding H. P., Ron D. (2000). Dynamic interaction of BiP and ER stress transducers in the unfolded-protein response. *Nature Cell Biology*.

[154] Suresh S. N., Verma V., Sateesh S., Clement J. P., Manjithaya R. (2018). Neurodegenerative diseases: model organisms, pathology and autophagy. *Journal of Genetics*.

[155] Koyano F., Okatsu K., Kosako H. (2014). Ubiquitin is phosphorylated by PINK1 to activate parkin. *Nature*.

[156] Tanaka K., Matsuda N. (2014). Proteostasis and neurodegeneration: the roles of proteasomal degradation and autophagy. *Biochimica et Biophysica Acta*.

[157] Alupei M. C., Maity P., Esser P. R. (2018). Loss of proteostasis is a pathomechanism in Cockayne syndrome. *Cell Reports*.

[158] Sacco R., Tamblyn L., Rajakulendran N., Bralha F. N., Tropepe V., Laposa R. R. (2013). Cockayne syndrome b maintains neural precursor function. *DNA Repair (Amst)*.

[159] Hossain M. M., DiCicco-Bloom E., Richardson J. R. (2015). Hippocampal ER stress and learning deficits following repeated pyrethroid exposure. *Toxicological Sciences*.

[160] Saito A., Kanemoto S., Kawasaki N. (2012). Unfolded protein response, activated by OASIS family transcription factors, promotes astrocyte differentiation. *Nature Communications*.

[161] Stone S., Lin W. (2015). The unfolded protein response in multiple sclerosis. *Frontiers in Neuroscience*.

[162] Traka M., Podojil J. R., McCarthy D. P., Miller S. D., Popko B. (2016). Oligodendrocyte death results in immune-mediated CNS demyelination. *Nature Neuroscience*.

[163] Clayton B. L. L., Popko B. (2016). Endoplasmic reticulum stress and the unfolded protein response in disorders of myelinating glia. *Brain Research*.

[164] Rinholm J. E., Hamilton N. B., Kessaris N., Richardson W. D., Bergersen L. H., Attwell D. (2011). Regulation of oligodendrocyte development and myelination by glucose and lactate. *The Journal of Neuroscience*.

[165] Saporta M. A. C., Shy B. R., Patzko A. (2012). *Mpz*R98C arrests Schwann cell development in a mouse model of early-onset Charcot–Marie–Tooth disease type 1B. *Brain*.

[166] Mollereau B., Rzechorzek N. M., Roussel B. D. (2016). Adaptive preconditioning in neurological diseases - therapeutic insights from proteostatic perturbations. *Brain Research*.

[167] Skibinski G., Hwang V., Ando D. M. (2017). Nrf2 mitigates LRRK2- and *α*-synuclein–induced neurodegeneration by modulating proteostasis. *Proceedings of the National Academy of Sciences of the United States of America*.

[168] Signer R. A. J., Morrison S. J. (2013). Mechanisms that regulate stem cell aging and life span. *Cell Stem Cell*.

[169] Cai X., Gao L., Teng L. (2015). Runx1 deficiency decreases ribosome biogenesis and confers stress resistance to hematopoietic stem and progenitor cells. *Cell Stem Cell*.

[170] Narla A., Ebert B. L. (2010). Ribosomopathies: human disorders of ribosome dysfunction. *Blood*.

[171] Khajuria R. K., Munschauer M., Ulirsch J. C. (2018). Ribosome levels selectively regulate translation and lineage commitment in human hematopoiesis. *Cell*.

[172] Mirabello L., Khincha P. P., Ellis S. R. (2017). Novel and known ribosomal causes of Diamond-Blackfan anaemia identified through comprehensive genomic characterisation. *Journal of Medical Genetics*.

[173] Miharada K., Karlsson G., Rehn M. (2011). Cripto regulates hematopoietic stem cells as a hypoxic-niche-related factor through cell surface receptor GRP78. *Cell Stem Cell*.

[174] Wey S., Luo B., Lee A. S. (2012). Acute inducible ablation of GRP78 reveals its role in hematopoietic stem cell survival, lymphogenesis and regulation of stress signaling. *PLoS One*.

[175] van Galen P., Kreso A., Mbong N. (2014). The unfolded protein response governs integrity of the haematopoietic stem-cell pool during stress. *Nature*.

[176] Kurisu J., Honma A., Miyajima H., Kondo S., Okumura M., Imaizumi K. (2003). MDG1/ERdj4, an ER-resident DnaJ family member, suppresses cell death induced by ER stress. *Genes to Cells*.

[177] Lai C. W., Otero J. H., Hendershot L. M., Snapp E. (2012). ERdj4 protein is a soluble endoplasmic reticulum (ER) DnaJ family protein that interacts with ER-associated degradation machinery. *The Journal of Biological Chemistry*.

[178] Chen T., Ozel D., Qiao Y. (2011). Chemical genetics identify eIF2*α* kinase heme-regulated inhibitor as an anticancer target. *Nature Chemical Biology*.

[179] Chen T., Takrouri K., Hee-Hwang S. (2013). Explorations of substituted urea functionality for the discovery of new activators of the heme-regulated inhibitor kinase. *Journal of Medicinal Chemistry*.

[180] Yefidoff-Freedman R., Fan J., Yan L. (2017). Development of 1-((1,4-*trans*)-4-aryloxycyclohexyl)-3-arylurea activators of heme-regulated inhibitor as selective activators of the eukaryotic initiation factor 2 alpha (eIF2*α*) phosphorylation arm of the integrated endoplasmic reticulum stress response. *Journal of Medicinal Chemistry*.

[181] Aktas B. H., Qiao Y., Ozdelen E. (2013). Small-molecule targeting of translation initiation for cancer therapy. *Oncotarget*.

[182] Chapple R. H., Hu T., Tseng Y. J. (2018). ER*α* promotes murine hematopoietic regeneration through the Ire1*α*-mediated unfolded protein response. *eLife*.

[183] Ho W.-M., Akyol O., Reis H. (2018). Autophagy after subarachnoid hemorrhage: can cell death be good?. *Current Neuropharmacology*.

[184] Liu F., Lee J. Y., Wei H. (2010). FIP200 is required for the cell-autonomous maintenance of fetal hematopoietic stem cells. *Blood*.

[185] Mortensen M., Watson A. S., Simon A. K. (2011). Lack of autophagy in the hematopoietic system leads to loss of hematopoietic stem cell function and dysregulated myeloid proliferation. *Autophagy*.

[186] Ito K., Hirao A., Arai F. (2006). Reactive oxygen species act through p38 MAPK to limit the lifespan of hematopoietic stem cells. *Nature Medicine*.

[187] Warr M. R., Kohli L., Passegué E. (2013). Born to survive: autophagy in hematopoietic stem cell maintenance. *Cell Cycle*.

[188] Moran-Crusio K., Reavie L. B., Aifantis I. (2012). Regulation of hematopoietic stem cell fate by the ubiquitin proteasome system. *Trends in Immunology*.

[189] Schmidt M. H. H., Dikic I. (2005). The Cbl interactome and its functions. *Nature Reviews Molecular Cell Biology*.

[190] Naujokat C., Fuchs D., Berges C. (2007). Adaptive modification and flexibility of the proteasome system in response to proteasome inhibition. *Biochimica et Biophysica Acta*.

[191] Tai-Nagara I., Matsuoka S., Ariga H., Suda T. (2014). Mortalin and DJ-1 coordinately regulate hematopoietic stem cell function through the control of oxidative stress. *Blood*.

[192] Miharada K., Sigurdsson V., Karlsson S. (2014). Dppa5 improves hematopoietic stem cell activity by reducing endoplasmic reticulum stress. *Cell Reports*.

[193] Rouault-Pierre K., Lopez-Onieva L., Foster K. (2013). HIF-2*α* protects human hematopoietic stem/progenitors and acute myeloid leukemic cells from apoptosis induced by endoplasmic reticulum stress. *Cell Stem Cell*.

[194] Mohrin M., Shin J., Liu Y. (2015). Stem cell aging. A mitochondrial UPR-mediated metabolic checkpoint regulates hematopoietic stem cell aging. *Science*.

[195] Kariminekoo S., Movassaghpour A., Rahimzadeh A., Talebi M., Shamsasenjan K., Akbarzadeh A. (2016). Implications of mesenchymal stem cells in regenerative medicine. *Artificial Cells, Nanomedicine, and Biotechnology*.

[196] Han J., Back S. H., Hur J. (2013). ER-stress-induced transcriptional regulation increases protein synthesis leading to cell death. *Nature Cell Biology*.

[197] Guo F. J., Xiong Z., Lu X., Ye M., Han X., Jiang R. (2014). ATF6 upregulates XBP1S and inhibits ER stress-mediated apoptosis in osteoarthritis cartilage. *Cellular Signalling*.

[198] Xiong Z., Jiang R., Zhang P., Han X., Guo F. J. (2015). Transmission of ER stress response by ATF6 promotes endochondral bone growth. *Journal of Orthopaedic Surgery and Research*.

[199] Zhang J., Weng Y., Liu X. (2013). Endoplasmic reticulum (ER) stress inducible factor cysteine-rich with EGF-like domains 2 (Creld2) is an important mediator of BMP9-regulated osteogenic differentiation of mesenchymal stem cells. *PLoS One*.

[200] Li Z., Wei H., Liu X., Hu S., Cong X., Chen X. (2010). LPA rescues ER stress-associated apoptosis in hypoxia and serum deprivation-stimulated mesenchymal stem cells. *Journal of Cellular Biochemistry*.

[201] Wu Y., Yang M., Fan J. (2014). Deficiency of osteoblastic Arl6ip5 impaired osteoblast differentiation and enhanced osteoclastogenesis via disturbance of ER calcium homeostasis and induction of ER stress-mediated apoptosis. *Cell Death & Disease*.

[202] Chen Y., Gao H., Yin Q. (2013). ER stress activating ATF4/CHOP-TNF-*α* signaling pathway contributes to alcohol-induced disruption of osteogenic lineage of multipotential mesenchymal stem cell. *Cellular Physiology and Biochemistry*.

[203] Mullick M., Sen D. (2018). The delta opioid peptide DADLE represses hypoxia-reperfusion mimicked stress mediated apoptotic cell death in human mesenchymal stem cells in part by downregulating the unfolded protein response and ROS along with enhanced anti-inflammatory effect. *Stem Cell Reviews*.

[204] van Berlo J. H., Molkentin J. D. (2014). An emerging consensus on cardiac regeneration. *Nature Medicine*.

[205] Cai C. L., Molkentin J. D. (2017). The elusive progenitor cell in cardiac regeneration: slip slidin’ away. *Circulation Research*.

[206] Klein D., Weißhardt P., Kleff V., Jastrow H., Jakob H. G., Ergün S. (2011). Vascular wall-resident CD44+ multipotent stem cells give rise to pericytes and smooth muscle cells and contribute to new vessel maturation. *PLoS One*.

[207] Whelan K. A., Chandramouleeswaran P. M., Tanaka K. (2017). Autophagy supports generation of cells with high CD44 expression via modulation of oxidative stress and Parkin-mediated mitochondrial clearance. *Oncogene*.

[208] Mekala S. R., Wörsdörfer P., Bauer J. (2018). *Circulation Research*.

[209] Xiao C., Wang K., Xu Y. (2018). Transplanted mesenchymal stem cells reduce autophagic flux in infarcted hearts via the exosomal transfer of mir-125b. *Circulation Research*.

[210] Sekine Y., Zyryanova A., Crespillo-Casado A., Fischer P. M., Harding H. P., Ron D. (2015). Stress responses. Mutations in a translation initiation factor identify the target of a memory-enhancing compound. *Science*.

[211] Das I., Krzyzosiak A., Schneider K. (2015). Preventing proteostasis diseases by selective inhibition of a phosphatase regulatory subunit. *Science*.

[212] Harding H. P., Zyryanova A. F., Ron D. (2012). Uncoupling proteostasis and development in vitro with a small molecule inhibitor of the pancreatic endoplasmic reticulum kinase, PERK. *The Journal of Biological Chemistry*.

[213] Sidrauski C., McGeachy A. M., Ingolia N. T., Walter P. (2015). The small molecule ISRIB reverses the effects of eIF2*α* phosphorylation on translation and stress granule assembly. *eLife*.

[214] Burwick N., Aktas B. H. (2017). The eIF2-alpha kinase HRI: a potential target beyond the red blood cell. *Expert Opinion on Therapeutic Targets*.

[215] Heneidi S., Simerman A. A., Keller E. (2013). Awakened by cellular stress: isolation and characterization of a novel population of pluripotent stem cells derived from human adipose tissue. *PLoS One*.

[216] Mahmoud E. E., Kamei N., Shimizu R. (2017). Therapeutic potential of multilineage-differentiating stress-enduring cells for osteochondral repair in a rat model. *Stem Cells International*.

[217] Yabuki H., Wakao S., Kushida Y., Dezawa M., Okada Y. (2018). Human multilineage-differentiating stress-enduring cells exert pleiotropic effects to ameliorate acute lung ischemia–reperfusion injury in a rat model. *Cell Transplantation*.

[218] Yamada Y., Wakao S., Kushida Y. (2018). S1P-S1PR2 axis mediates homing of Muse cells into damaged heart for long-lasting tissue repair and functional recovery after acute myocardial infarction. *Circulation Research*.

[219] Uchida H., Niizuma K., Kushida Y. (2017). Human Muse cells reconstruct neuronal circuitry in subacute lacunar stroke model. *Stroke*.

[220] Uchida N., Kushida Y., Kitada M. (2017). Beneficial effects of systemically administered human Muse cells in adriamycin nephropathy. *Journal of the American Society of Nephrology*.

[221] Iseki M., Kushida Y., Wakao S. (2017). Muse cells, nontumorigenic pluripotent-like stem cells, have liver regeneration capacity through specific homing and cell replacement in a mouse model of liver fibrosis. *Cell Transplantation*.

[222] Shimamura N., Kakuta K., Wang L. (2017). Neuro-regeneration therapy using human Muse cells is highly effective in a mouse intracerebral hemorrhage model. *Experimental Brain Research*.

[223] Chong J. J. H., Chandrakanthan V., Xaymardan M. (2011). Adult cardiac-resident MSC-like stem cells with a proepicardial origin. *Cell Stem Cell*.

[224] Hsu Y. C., Fuchs E. (2012). A family business: stem cell progeny join the niche to regulate homeostasis. *Nature Reviews Molecular Cell Biology*.

[225] Spradling A., Drummond-Barbosa D., Kai T. (2001). Stem cells find their niche. *Nature*.

[226] Boni A., Urbanek K., Nascimbene A. (2008). Notch1 regulates the fate of cardiac progenitor cells. *Proceedings of the National Academy of Sciences of the United States of America*.

[227] Urbanek K., Cabral-da-Silva M. C., Ide-Iwata N. (2010). Inhibition of notch1-dependent cardiomyogenesis leads to a dilated myopathy in the neonatal heart. *Circulation Research*.

[228] Wang D.‐. Y., Abbasi C., el-Rass S. (2014). Endoplasmic reticulum resident protein 44 (ERp44) deficiency in mice and zebrafish leads to cardiac developmental and functional defects. *Journal of the American Heart Association*.

[229] Maass A., Leinwand L. A. (2001). A role for calreticulin in the adult heart?. *The Journal of Clinical Investigation*.

[230] Rauch F., Prud'homme J., Arabian A., Dedhar S., St-Arnaud R. (2000). Heart, brain, and body wall defects in mice lacking calreticulin. *Experimental Cell Research*.

[231] Judge L. M., Perez-Bermejo J. A., Truong A. (2017). A BAG3 chaperone complex maintains cardiomyocyte function during proteotoxic stress. *JCI Insight*.

[232] Cornelison D., Perdiguero E. (2017). Muscle stem cells: a model system for adult stem cell biology. *Methods in Molecular Biology*.

[233] Relaix F., Zammit P. S. (2012). Satellite cells are essential for skeletal muscle regeneration: the cell on the edge returns centre stage. *Development*.

[234] Cheung T. H., Rando T. A. (2013). Molecular regulation of stem cell quiescence. *Nature Reviews Molecular Cell Biology*.

[235] Montarras D., L'Honore A., Buckingham M. (2013). Lying low but ready for action: the quiescent muscle satellite cell. *The FEBS Journal*.

[236] Cheung T. H., Quach N. L., Charville G. W. (2012). Maintenance of muscle stem-cell quiescence by microRNA-489. *Nature*.

[237] Crist C. G., Montarras D., Buckingham M. (2012). Muscle satellite cells are primed for myogenesis but maintain quiescence with sequestration of Myf5 mRNA targeted by microRNA-31 in mRNP granules. *Cell Stem Cell*.

[238] Zismanov V., Chichkov V., Colangelo V. (2016). Phosphorylation of eIF2*α* is a translational control mechanism regulating muscle stem cell quiescence and self-renewal. *Cell Stem Cell*.

[239] Garcia-Prat L., Martinez-Vicente M., Munoz-Canoves P. (2016). Autophagy: a decisive process for stemness. *Oncotarget*.

[240] García-Prat L., Martínez-Vicente M., Perdiguero E. (2016). Autophagy maintains stemness by preventing senescence. *Nature*.

[241] Fiacco E., Castagnetti F., Bianconi V. (2016). Autophagy regulates satellite cell ability to regenerate normal and dystrophic muscles. *Cell Death and Differentiation*.

[242] Fortini P., Ferretti C., Iorio E. (2016). The fine tuning of metabolism, autophagy and differentiation during in vitro myogenesis. *Cell Death & Disease*.

[243] Fortini P., Iorio E., Dogliotti E., Isidoro C. (2016). Coordinated metabolic changes and modulation of autophagy during myogenesis. *Frontiers in Physiology*.

[244] Garcia-Prat L., Munoz-Canoves P., Martinez-Vicente M. (2016). Dysfunctional autophagy is a driver of muscle stem cell functional decline with aging. *Autophagy*.

[245] Sousa-Victor P., Gutarra S., García-Prat L. (2014). Geriatric muscle stem cells switch reversible quiescence into senescence. *Nature*.

[246] Yousefi M., Li L., Lengner C. J. (2017). Hierarchy and plasticity in the intestinal stem cell compartment. *Trends in Cell Biology*.

[247] Krausova M., Korinek V. (2014). Wnt signaling in adult intestinal stem cells and cancer. *Cellular Signalling*.

[248] Strubberg A. M., Liu J., Walker N. M. (2018). Cftr modulates Wnt/*β*-catenin signaling and stem cell proliferation in murine intestine. *Cellular and Molecular Gastroenterology and Hepatology*.

[249] Heijmans J., van Lidth de Jeude J. F., Koo B. K. (2013). ER stress causes rapid loss of intestinal epithelial stemness through activation of the unfolded protein response. *Cell Reports*.

[250] Wang L., Zeng X., Ryoo H. D., Jasper H. (2014). Integration of UPRER and oxidative stress signaling in the control of intestinal stem cell proliferation. *PLoS Genetics*.

[251] Niederreiter L., Fritz T. M. J., Adolph T. E. (2013). ER stress transcription factor Xbp1 suppresses intestinal tumorigenesis and directs intestinal stem cells. *The Journal of Experimental Medicine*.

[252] Cao S. S., Song B., Kaufman R. J. (2012). PKR protects colonic epithelium against colitis through the unfolded protein response and prosurvival signaling. *Inflammatory Bowel Diseases*.

[253] Fenelon J. C., Lefevre P. L., Banerjee A., Murphy B. D. (2017). Regulation of diapause in carnivores. *Reproduction in Domestic Animals*.

[254] Bulut-Karslioglu A., Biechele S., Jin H. (2016). Inhibition of mTOR induces a paused pluripotent state. *Nature*.

